# Effects of handedness on brain oscillatory activity during imagery and execution of upper limb movements

**DOI:** 10.3389/fpsyg.2023.1161613

**Published:** 2023-06-13

**Authors:** Melissa Lajtos, Luis Alberto Barradas-Chacón, Selina Christin Wriessnegger

**Affiliations:** ^1^Institute of Neural Engineering, Graz University of Technology, Graz, Austria; ^2^Medical Image and Signal Processing (MEDISIP), Department of Electronics and Information Systems (ELIS), Ghent University, Ghent, Belgium

**Keywords:** EEG, motor imagery, ERD, ERS, motor execution, handedness

## Abstract

Brain activation during left- and right-hand motor imagery is a popular feature used for brain–computer interfaces. However, most studies so far have only considered right-handed participants in their experiments. This study aimed to investigate how handedness influences brain activation during the processes of imagining and executing simple hand movements. EEG signals were recorded using 32 channels while participants repeatedly squeezed or imagined squeezing a ball using their left, right, or both hands. The data of 14 left-handed and 14 right-handed persons were analyzed with a focus on event-related desynchronization/synchronization patterns (ERD/S). Both handedness groups showed activation over sensorimotor areas; however, the right-handed group tended to display more bilateral patterns than the left-handed group, which is in contrast to earlier research results. Furthermore, a stronger activation during motor imagery than during motor execution could be found in both groups.

## 1. Introduction

The prevalence of left-handedness in people is estimated to be 10.6% overall but ranges from 9.3 to 18.1%. Those variations can partially be attributed to participant characteristics, such as sex and ancestry (Papadatou-Pastou et al., 2020). Although handedness is the clearest example of behavioral lateralization in humans, it is still debatable how this preference is reflected in the motor organization in the brain (Crotti et al., 2022). Researchers have obtained various results in recent decades when investigating neural activation during unilateral hand movements but have regularly found activation of the primary motor and sensory areas. Some studies found bilateral activation within motor-related brain areas, with some differences in activation intensities based on handedness (Kim et al., 1993; Singh et al., 1998; Baraldi et al., 1999). A study by Dassonville et al. (1997) found a greater volume of activation in the contralateral motor cortex when using the dominant hand. They also found a separate relationship between the degree of handedness and the extent of functional lateralization in the motor cortex. Some studies suggest that left-handers recruit a more bilateral network during hand or finger movements than right-handed people (Martin et al., 2011; Tzourio-Mazoyer et al., 2015). Kawashima et al. (1997) suggested that during unilateral finger movements hemispheric asymmetry exists only in the functional activation of the pre-motor area in left-handers but not in the primary motor area and the supplementary motor area. While a number of studies found differences, which can be attributed to handedness in the neural correlates of motor execution (ME), fewer studies focused on motor imagery (MI). Zapała et al. investigated the effects of handedness on sensorimotor rhythms (SMR) during MI tasks. Their results showed that left-handed individuals present weaker SMR suppression in the alpha band during left-hand MI (Zapała et al., 2020). In another study, they found results indicating that left- and right-handers imagine movement differently, with left-handed individuals focusing more on visual experience (Zapała et al., 2021). A recent fMRI study by Crotti et al. (2022) investigated neural correlates during ME and MI of simple hand movements in left- and right-handed individuals. For ME, they found that left-handed participants recruited a spread bilateral network, while right-handers showed a more lateralized activity. For MI on the other hand, they found that for both groups the strongest activation could be found in the ipsilateral hemisphere.

Pfurtscheller and Neuper (2001) already showed that brain activity changes related to MI can serve as useful control signals for brain–computer interfaces (BCIs). Since then, research on BCIs has become more interesting for a broader community of researchers and users, as there has been a shift from applications for individuals with motor impairments to healthy users (Allison et al., 2007). However, most BCI studies only considered right-handed participants, as they represent a large majority of the population. This is only one good reason why it is important to further investigate what effects handedness has on neurophysiological patterns used for BCI.

This study aims to further investigate differences in neural correlates between left- and right-handed people during simple hand motor imagery and execution tasks. We will specifically look for differences in event-related desynchronization (ERD), which is said to reflect an activation of the brain area concerned and event-related synchronization (ERS), or deactivation of concerned brain areas, as introduced by Pfurtscheller and Da Silva (1999). ERD/S values are commonly used in recent studies (Wriessnegger et al., 2018; Zapała et al., 2021; Grazia et al., 2022) and are popular as features in MI-based BCI systems (Pfurtscheller and Neuper, 2006; Hwang et al., 2013; Wierzgała et al., 2018; Singh et al., 2021). Here, ME is used as a control condition as it is already well known that MI brain activity parallels that of ME (Kraeutner et al., 2014), and the underlying neural correlates are quite clear (Pfurtscheller, 2000; Neuper et al., 2006; Nakayashiki et al., 2014). We hypothesized that handedness will have an influence on the brain (de)activation patterns during both the execution and imagery of simple hand movements. In particular, we expected that left-handed participants will show more bilateral activation patterns than right-handed participants in both the alpha and beta band and in both ME and MI based on previous studies.

Furthermore, we expect that (1) ME and MI will induce spatially overlapping activation patterns; (2) ERD will be found over sensorimotor areas during both ME and MI; (3) ERD will be stronger in the contralateral hemisphere of hand movement.

## 2. Materials and methods

### 2.1. Participants

Thirty-one healthy participants took part in the study. The handedness (left vs. right) of each participant was assessed using the Edinburgh Handedness Inventory (EHI) (Oldfield, 1971). In cases where the result of the EHI indicated neither left nor right handedness (i.e., the laterality index was within the middle decile), the participants also completed the hand dominance test (HDT) (Steingrüber and Lienert, 1971); three participants were excluded from data analysis—one due to not being identifiable as either a strict left- or right-handed person based on their handedness assessment, and also two right-handed females due to poor EEG data quality (arising partially due to high impedances). Thus, 28 participants between 19 and 33 years of age were considered for data analysis, half of whom were left handed (age mean = 24.7 years; SD = 4.01), the other half being right handed (age mean = 26.8 years; SD = 3.47). The groups were also balanced in terms of sex. To assess their ability to perform kinesthetic MI, participants filled out the kinesthetic part of the Vividness of Motor Imagery Questionnaire II (VMIQ-2) (Roberts et al., 2008) right after the actual experiment. All participants were informed about the purpose of the study before giving their written consent. The study was approved by the local ethics committee (Medical University of Graz) and was performed in accordance with the ethical standards of the Declaration of Helsinki.

### 2.2. Data acquisition

#### 2.2.1. Participant preparation

Each participant was given a thorough explanation of the experiment and was able to ask questions about it before the experiment started. To get familiar with the tasks, they were handed two test balls and instructed to squeeze them repeatedly, using only their left, right, or both hands. They were asked to squeeze the balls at their own pace and intensity (gradually increasing and decreasing pressure, rather than short “pulses” of squeezing), but to keep those consistent throughout the experiment, and to sync the movements of both hands in BOTH conditions.

An explanation of the concept of MI and its variants (internal MI, external MI, kinesthetic MI) was given. The participant then imagined squeezing the balls repeatedly using kinesthetic imagery for a few seconds to become familiar with the concept. They were also asked to use only that form of MI during the MI runs. Finally, they were shown the images that served as cues in the experimental paradigm, emphasizing the three different conditions (LEFT, RIGHT, BOTH).

After electrode placement (as described in Section 2.2.4), the participant was seated in an armchair inside a dimly lit measurement box. In front of the participant was a computer screen that was used to present the visual input from the experimental paradigm to the participant. During the experiment, the participants rested their arms on the armrests with their palms pointing upwards. They were told to avoid unnecessary movements during the presentation of a fixation cross or a cue. Once the participants no longer had any more questions, the paradigm and the EEG recording were started. Before each run, the participant was told whether to perform motor execution or imagination during the upcoming run. Accordingly, the balls were taken away from or given to the participant.

The procedures for MI and ME were almost the same, with the only difference being that during MI the participant was no longer holding the balls and only imagining the hand movements.

#### 2.2.2. Experimental setup

All experiments were conducted inside facilities of Graz University of Technology. Each participant stayed in the same room for the whole duration of their respective experimental session. The room contained a number of desks and chairs as well as one measurement box. One wall of the room had windows through which natural light could enter. The room was thoroughly ventilated before the participants arrived.

During preparation, the participant sat at one of the desks in an office chair. The VMIQ-2 was filled out on the same desk digitally on a laptop. The measurement box—designed as a Faraday cage—that was used for EEG measurements was dimly lit and contained an office armchair, a small table with a screen on top, and the measurement devices (amplifier etc.). The table with the computer screen (diagonal length: 61 cm) was placed ~ 1.3 m in front of the chair. The box had a small tinted window in the door, allowing the experimenter to observe the participant. Participants were asked to keep electronic devices such as phones and smartwatches outside the measurement box.

**Figure 1:**
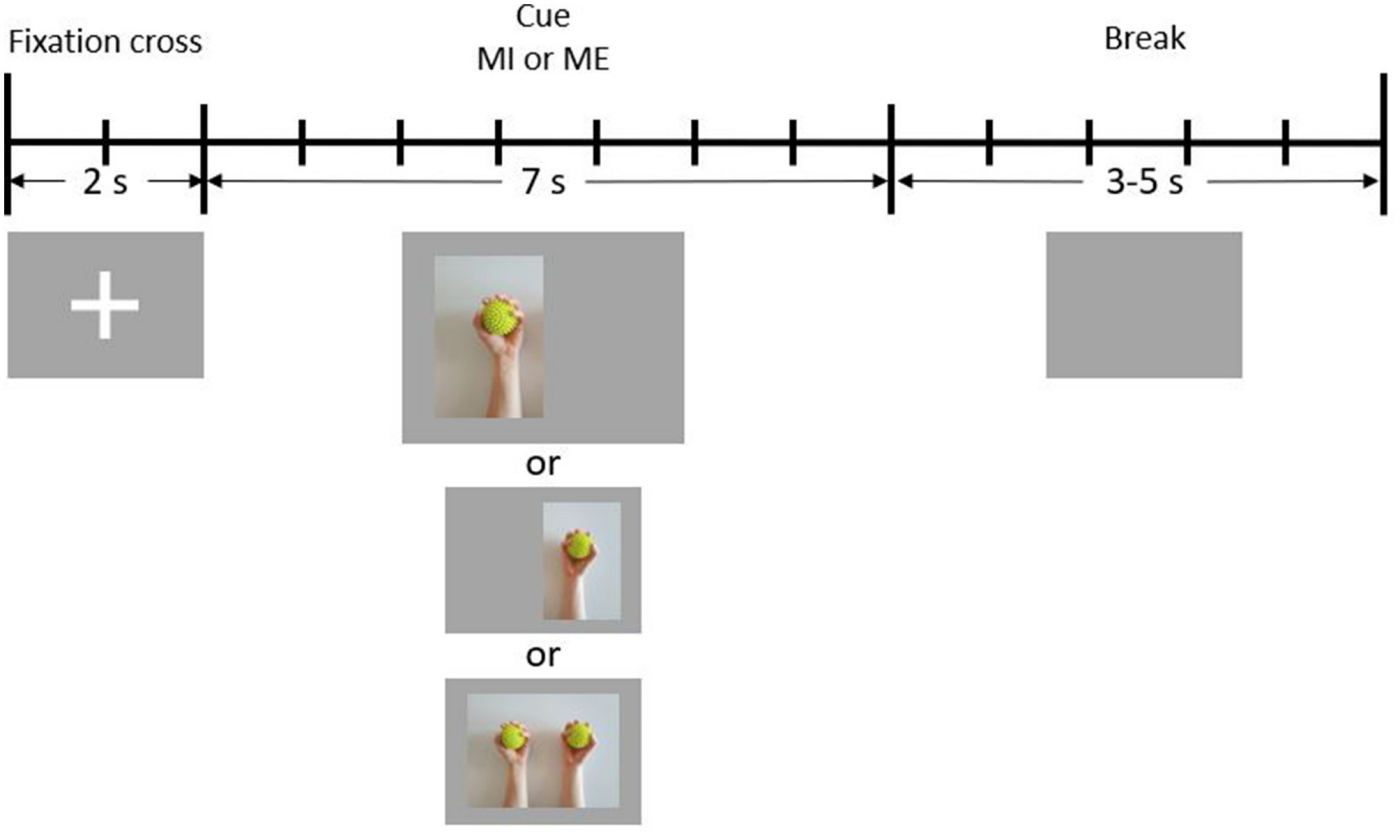
Schematic illustration of one trial. During the first 2 s of each trial, a fixation cross is presented to the participant (reference period). In the following 7 s, a cue is presented (condition period). Finally, there is a break period of 3–5 s during which the screen is blank.

The balls used during the ME paradigm were massage balls with spikes on the surface. They are visible in the cue images shown in Figure 1. The balls were disinfected after every session.

#### 2.2.3. Study paradigm

The paradigm was created using PsychoPy (Peirce et al., 2019) and was almost identical to the one used in a previous fMRI study by Crotti et al. (2022). Participants had to perform an execution and imagery task (tasks ME and MI) of repetitively squeezing a ball using their left, right, or both hands (conditions LEFT, RIGHT, and BOTH). The paradigm consisted of six blocks, with three blocks being purely ME and the other three purely MI. Blocks were completed in alternating order, with approximately half of the participants starting with an MI run. Break periods were given between runs, during which the recording was stopped. Besides giving participants an opportunity to rest, these periods were also used for asking the participants about their experiences regarding the preceding run. After the participants stated that they felt ready, the experiment continued with the next block. Participants were told before each run which task (ME or MI) they had to perform.

One run took ~6.5 min and consisted of a short pre-run period, 30 trials (containing a BOTH, LEFT, or RIGHT condition) and a black screen (end of trial). During the pre-run period, a text screen presented general instructions (i.e., “avoid unnecessary movements”) to the participant. This screen was followed by 5 s of an empty screen, after which the first trial started. Trials were randomized using 10 trials per condition within each run.

Figure 1 depicts the timeline of a single trial. Each trial started with 2 s of reference time, during which a fixation cross was visible on the screen. Participants were instructed to sit still during this time period. Immediately after the fixation cross had disappeared, one of three cues indicating the condition to be performed was presented for 7 s. The cue for the condition BOTH appeared in the center of the screen, while the cue for LEFT was shifted slightly to the left, and the cue for RIGHT was shifted slightly to the right. During this period, either the action or the imagination of repetitive squeezing one or both balls had to be performed. At the end of each trial, a jitter interval of 3–5 s resting time (break) took place, during which the screen was blank. Participants were instructed to avoid unnecessary movements (e.g., blinking, teeth clenching) during the reference and the condition periods.

#### 2.2.4. EEG acquisition

**Figure 2:**
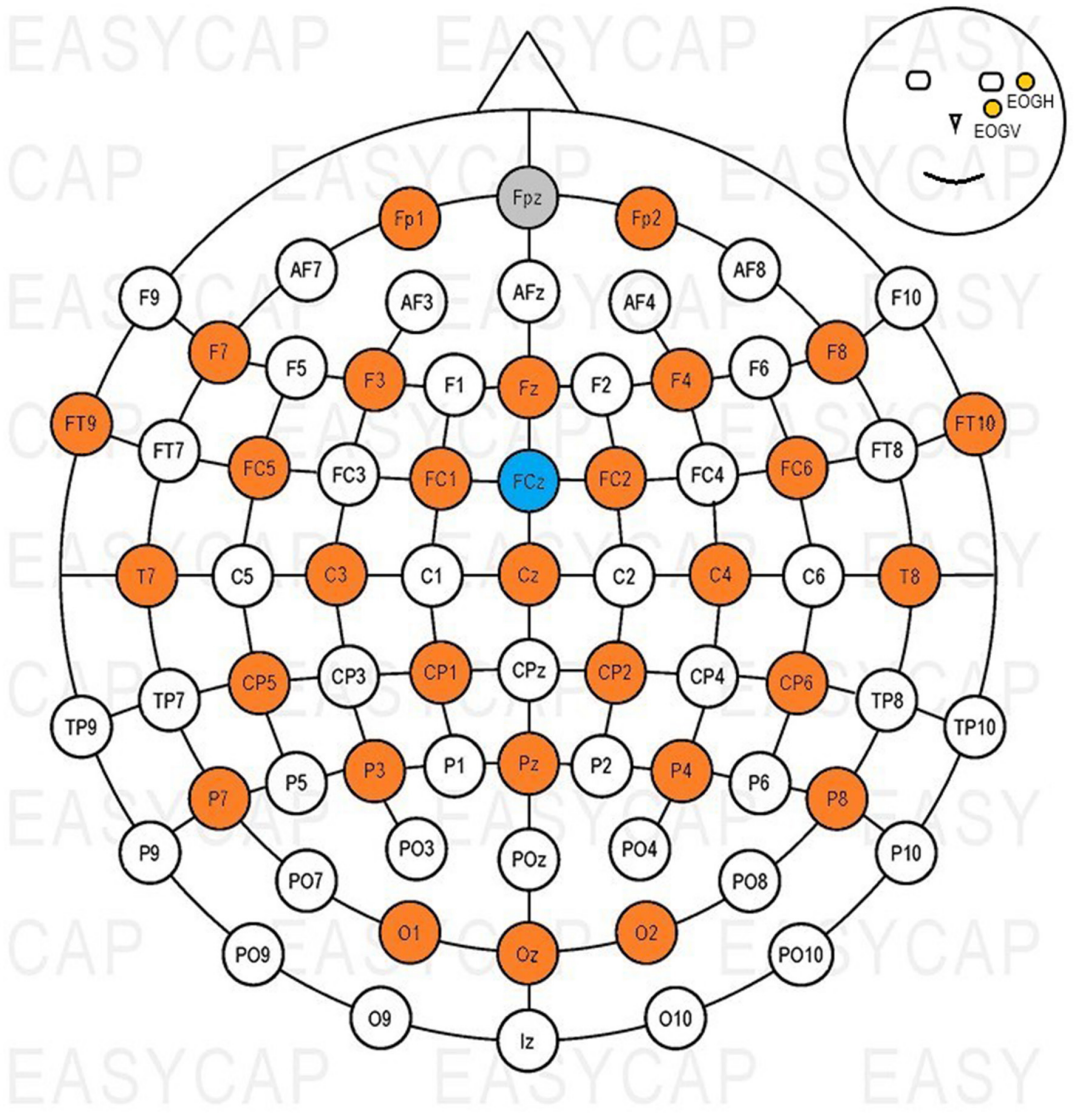
Electrode positions used with the EasyCAP. Gray: GND, blue: REF, orange: EEG channels, yellow: EOG channels.

Thirty-two active Ag-AgCl scalp electrodes (actiCAP slim electrodes, Brain Products GmbH), were used to record signals, and in addition to this, both a ground (GND) and a reference (REF) electrodes were used. An elastic cap (EasyCAP with actiCAP snap holders, Brain Products GmbH) was used to position the electrodes on the scalp according to the international 10–20 system (Jasper, 1958). Figure 2 shows the electrode layout of the cap, with colors highlighting the positions used during measurements. The ground electrode was placed on position Fpz, the reference on FCz; two electrodes were used to record eye activity. One was placed next to the outer canthus (EOGH) of and the other below (EOGV) the left eye (Figure 2).

Attempts were made to keep the impedance level below 20 kΩ throughout the whole measurement sessions, although up to three electrodes with impedances between 20 and 60 kΩ were tolerated. Impedances were checked regularly between measurement blocks. When increased impedances were noticed, the target level was restored before starting the next block. When an impedance exceeded 60 kΩ, the block and electrode(s) in question were noted down to be given dedicated attention during later pre-processing. There were two blocks in total where an impedance above 60 kΩ was observed.

The BrainVision Recorder application was used to visualize the recorded signals during measurements, while its Remote Data Access (RDA) was used to send data to Lab Streaming Layer (LSL) (Kothe et al., 2019) at a sampling rate of 500 Hz. LSL LabRecorder collected both the EEG time series from the BrainVision Recorder and the markers sent from PsychoPy and recorded the data of each run (six per participant) in a separate XDF file.

### 2.3. Data analysis

#### 2.3.1. EEG preprocessing

All data processing was performed in Python 3.10. Raw data were read from XDF files and preprocessed using MNE (Gramfort et al., 2013) version 1.0.3 (Larson et al., 2022). Data from ME and MI runs were processed separately.

Time series were inspected visually. One participant presented a flat channel (FT9) in two runs, which was interpolated using spherical spline interpolation (Perrin et al., 1989). Next, all EEG channels were re-referenced to a virtual average reference. Independent component analysis (ICA) using the Fast ICA approach (Hyvarinen, 1999) was used to manually remove components that resembled blinking and eye saccades. In some cases, additional components that seemed to contain noise specific to a single especially noisy channel (mostly T7 and T8) were selected for removal to improve the quality of those channels. Afterward, the data were filtered between 1 and 40 Hz using a non-causal FIR bandpass filter. Once all these steps were finished, the signals were again inspected visually for quality.

Next, epochs were created from 2 s before to 7 s after cue onset (defined as time point 0), resulting in a baseline interval from −2 to 0 s. Epochs were rejected based on the maximum EEG peak-to-peak amplitude, i.e., the absolute difference between the lowest and the highest signal value, within a said epoch. Different thresholds were used between participants, which were chosen after visual inspection of the signals (e.g., bigger thresholds were chosen for participants who showed particularly strong alpha waves). The lowest threshold, which was used for 23 of 31 participants, was 120 μV, while in the remaining cases mostly a threshold of 150 μV was used.

#### 2.3.2. ERD/S analysis

ERD/S values (Pfurtscheller and Da Silva, 1999) were used for statistical analysis as well as for the creation of ERD/S topoplots. The equation for calculating the ERD/S value of one epoch is given by


(1) $$\begin{array}{l} ERDS\;=\;\frac{A\;-\;R}{R} \end{array}$$

with A being the average bandpower during the activation phase of the epoch and R being the average bandpower during the baseline period.

The detailed process of obtaining these values for the purpose of this study was the following: All epochs were bandpass filtered [8–13 Hz for the alpha band, 16–24 Hz for the beta band; in accordance with a previous study (Wriessnegger et al., 2018)] using a non-causal FIR filter. The time series were squared to obtain continuous band powers. Subsequently, Equation 1 was used to calculate the ERD/S values for each epoch and channel, for which the reference period was taken by the −1.5 to 0 s interval, while the activation period consisted of the time window 2–6 s post-cue. The resulting value was multiplied by 100 to obtain a percentage. For each participant, task (ME, MI), and condition (LEFT, RIGHT, BOTH), the mean value was calculated over trials.

Generating topographic plots with grand averages over participant groups required one more step, namely computing the mean for each group. The obtained values were passed to MNE's function “mne.viz.plot topomap()” to create the final topoplots. For every participant/group and task, one figure was created which contains one topoplot per condition and frequency band.

**Figure 3:**
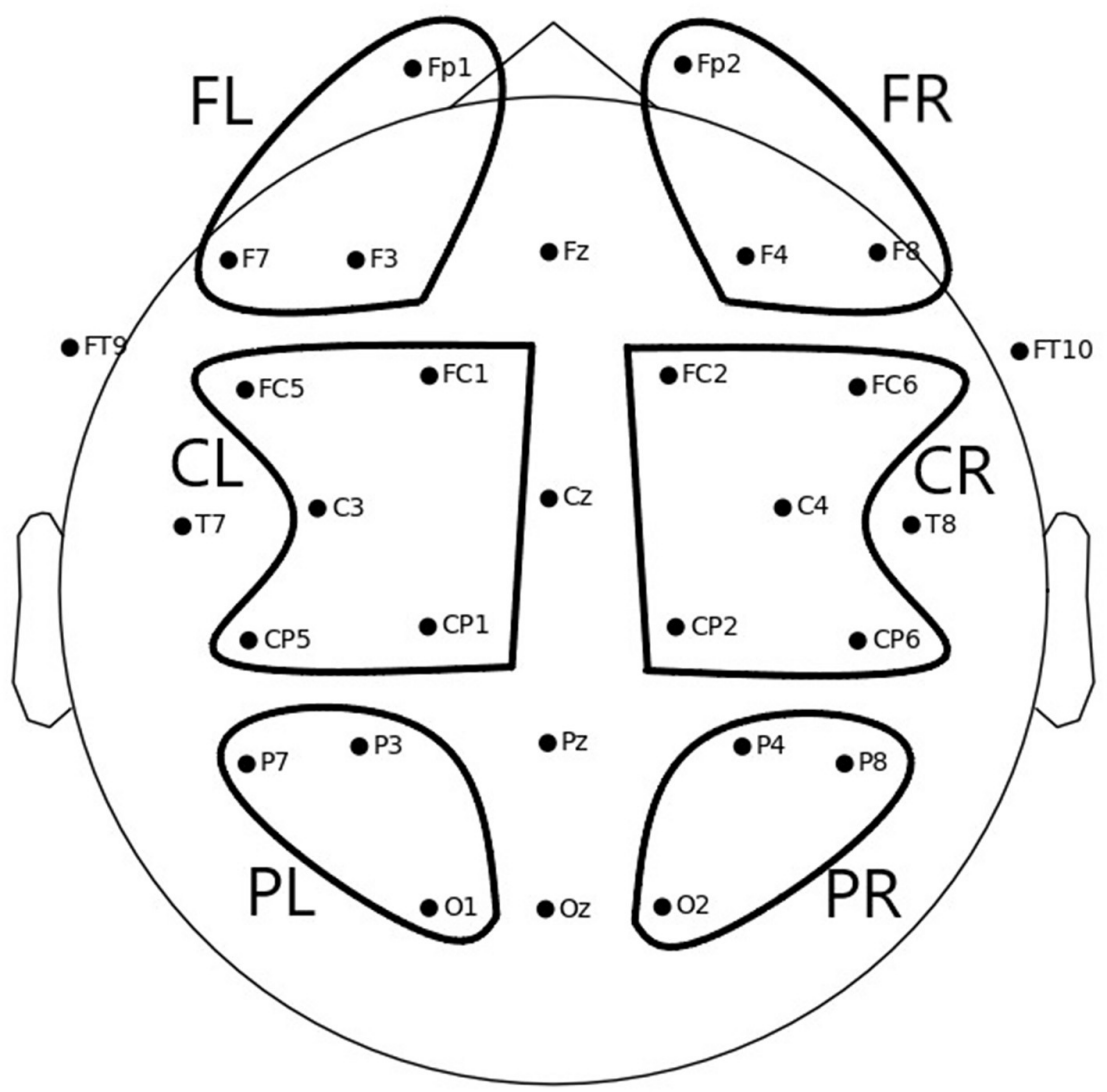
Electrode positions and six ROIs over which average ERD/S values were computed to be used for statistical analysis: FL, frontal left; FR, frontal right; CL, central left; CR, central right; PL, parietal left; PR, parietal right.

To obtain the values used in the statistical analysis, the mean ERD/S values per channel and condition were averaged per region of interest (ROI) for each participant. Six different ROIs were defined, as depicted in Figure 3:

- Frontal left (FL): Fp1, F3, F7
- Frontal right (FR): Fp2, F4, F8
- Central left (CL): FC1, FC5, C3, CP1, CP5
- Central right (CR): FC2, FC6, C4, CP2, CP6
- Parietal left (PL): P3, P7, O1
- Parietal right (PR): P4, P8, O2

Time-frequency ERD/S maps were also generated using a different procedure. A spectrogram was computed with consecutive Fourier transforms using SciPy's (Virtanen et al., 2020) function “scipy.signal.spectrogram()”, using a Tukey window, a segment length of 250, and 225 points of overlap between segments. For the scaling parameter, the power spectrum was selected. Almost the whole time series of the epochs were used, namely −1.5 to 7 s. Frequencies from 1 to 35 Hz were considered. The results were averaged across channels per ROI.

#### 2.3.3. Statistical analysis

Twenty-eight of the 31 participating persons were considered for analysis. Taking these into consideration, 163 epochs were rejected during pre-processing out of 5,040 in total (180 per participant), leaving 4,877 clean epochs, which corresponds to 96.77%.

To investigate the potential influence of handedness and the condition on the ERD/S patterns, four 6 × 3 repeated-measures analyses of variance (RMANOVAs) were performed using the software Jamovi (Love et al., 2021). ERD/S values were analyzed for each task (ME, MI) and frequency band (alpha, beta) separately considering the variables ROI (six levels: FL, FR, CL, CR, PL, PR) and condition (three levels: BOTH, LEFT, RIGHT) as within-subject variables. Between-subject factors were given by Handedness (left, right) and Sex (female, male).

In each of the four ANOVAs, a Greenhouse–Geisser correction was applied as the data showed a lack of sphericity. For *post-hoc* analysis, Tukey-corrected *p*-values were used to control for multiple comparisons. An α-level of 0.05 was used to indicate statistical significance.

## 3. Results

### 3.1. Quantitative results

The results of the EHI for left-handed participants were as follows: mean = −77.9, STD = 20.6, ranging from −100 to −20. For right-handed participants, they were as follows: mean = 86.8, STD = 11.5, ranging from 60 to 100. The possible outcome of the EHI spans a ranging from −100, indicating strong left-handedness, to +100, indicating strong right-handedness. The one participant scoring −20 on the EHI also completed the HDT, which provides a behavioral measure of hand dominance. They reached an overall score of −18, which confirmed their self-reported left-handedness.

The results of the VMIQ-2 for left-handed participants were as follows: mean = 24.7, STD = 5.59, ranging from 15 to 35. For right-handed participants, these were as follows: mean = 25.2, STD = 6.27, ranging from 15 to 35. In this test, results of between 12 and 60 are possible, where a lower score indicates a better ability to perform motor imagery.

### 3.2. Brain activation

Although topographical plots were created for every single participant, only the grand averages over the handedness groups (left/right) will be shown here. No separation by sex is made, as no statistically significant differences were found between males and females.

**Figure 4:**
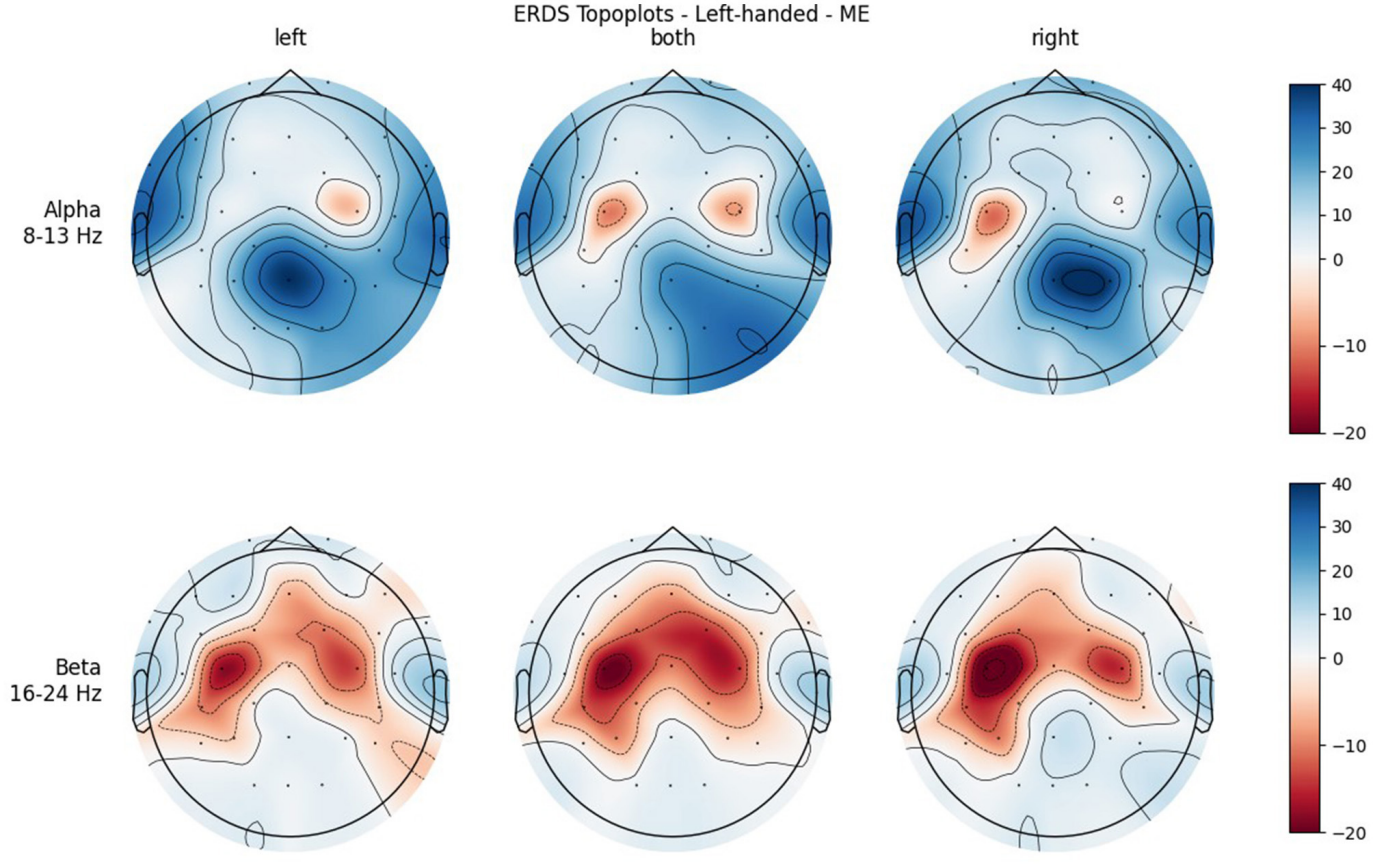
Topographical plots of mean ERD/S values (percentages relative to baseline interval) taken over all trials performed by left-handed participants during ME. Top row: alpha band (8–13 Hz); bottom row: beta band (16–24 Hz). Columns from left to right correspond to the three different conditions of left hand, both hands, and right hand movement. Red colors indicate ERD; blue colors, ERS.

**Figure 5:**
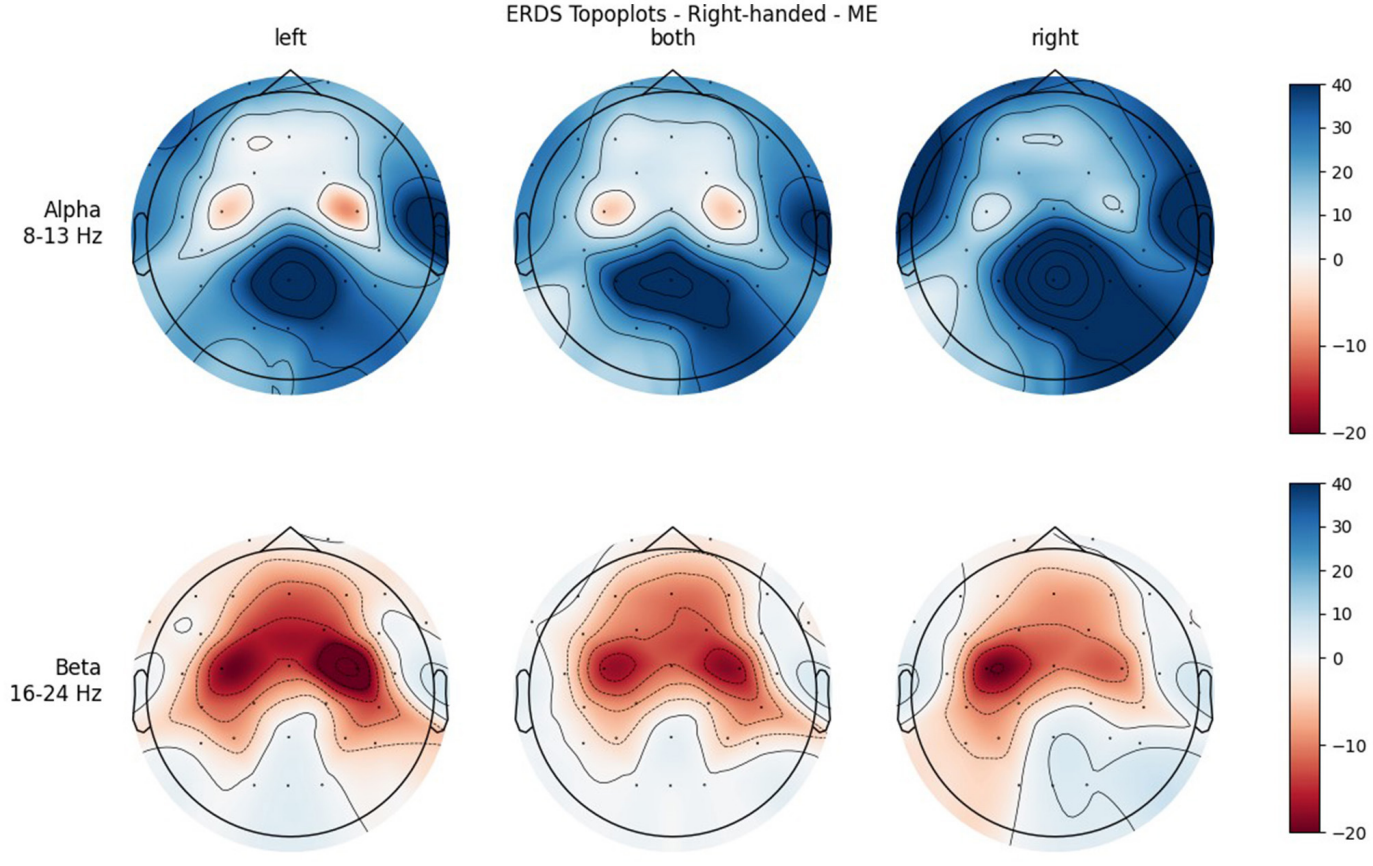
Topographical plots of mean ERD/S values (percentages relative to baseline interval) taken over all trials performed by right-handed participants during MI. Top row: alpha band (8–13 Hz); bottom row: beta band (16–24 Hz). Columns from left to right correspond to the three different conditions of left hand, both hands, and right hand movement. Red colors indicate ERD; blue colors, ERS.

**Figure 6:**
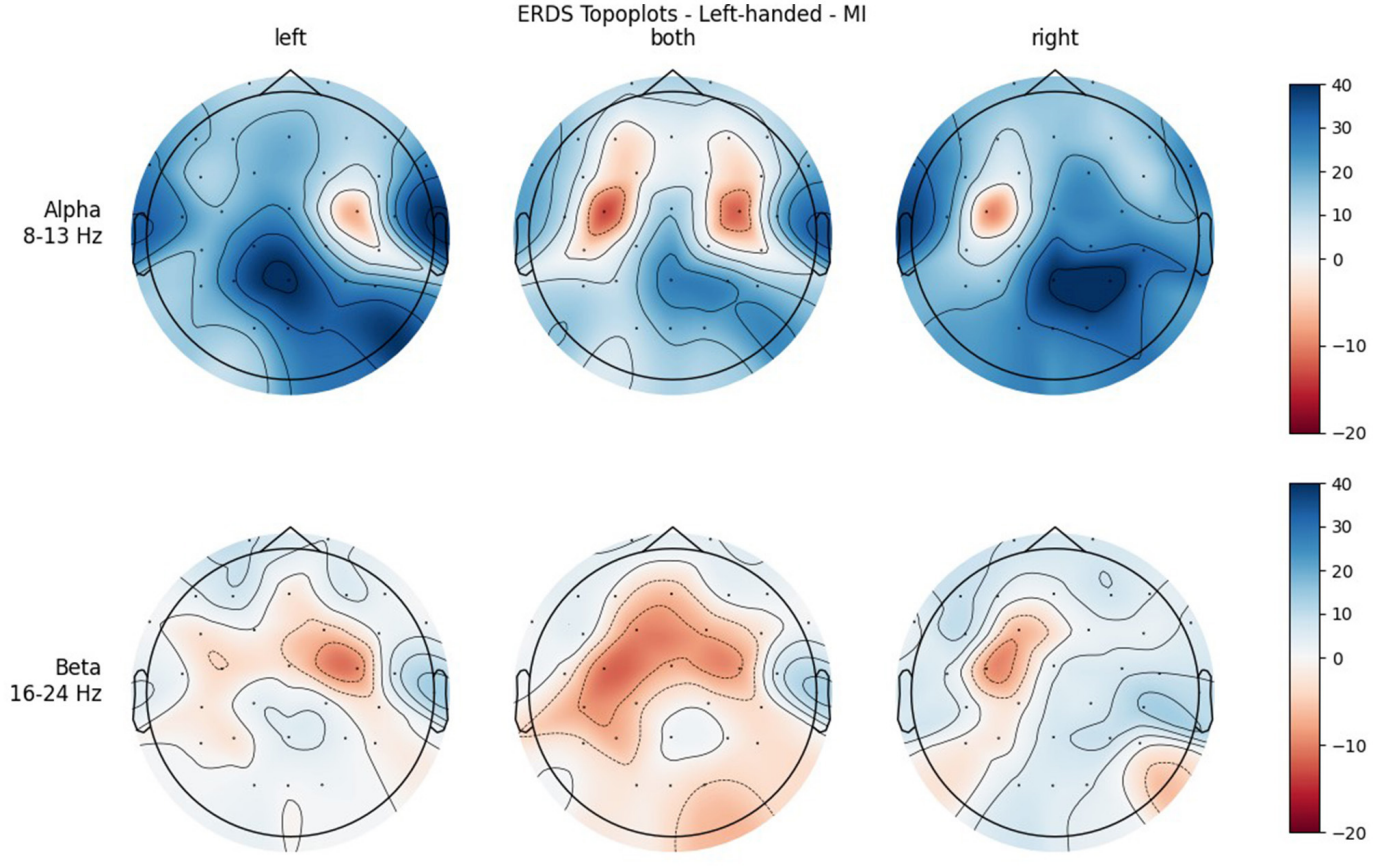
Topographical plots of mean ERD/S values (percentages relative to baseline interval) taken over all trials performed by left-handed participants during MI. Top row: alpha band (8–13 Hz), bottom row: beta band (16–24 Hz). Columns from left to right correspond to the three different conditions of left hand, both hands, and right hand movement. Red colors indicate ERD; blue colors, ERS.

**Figure 7:**
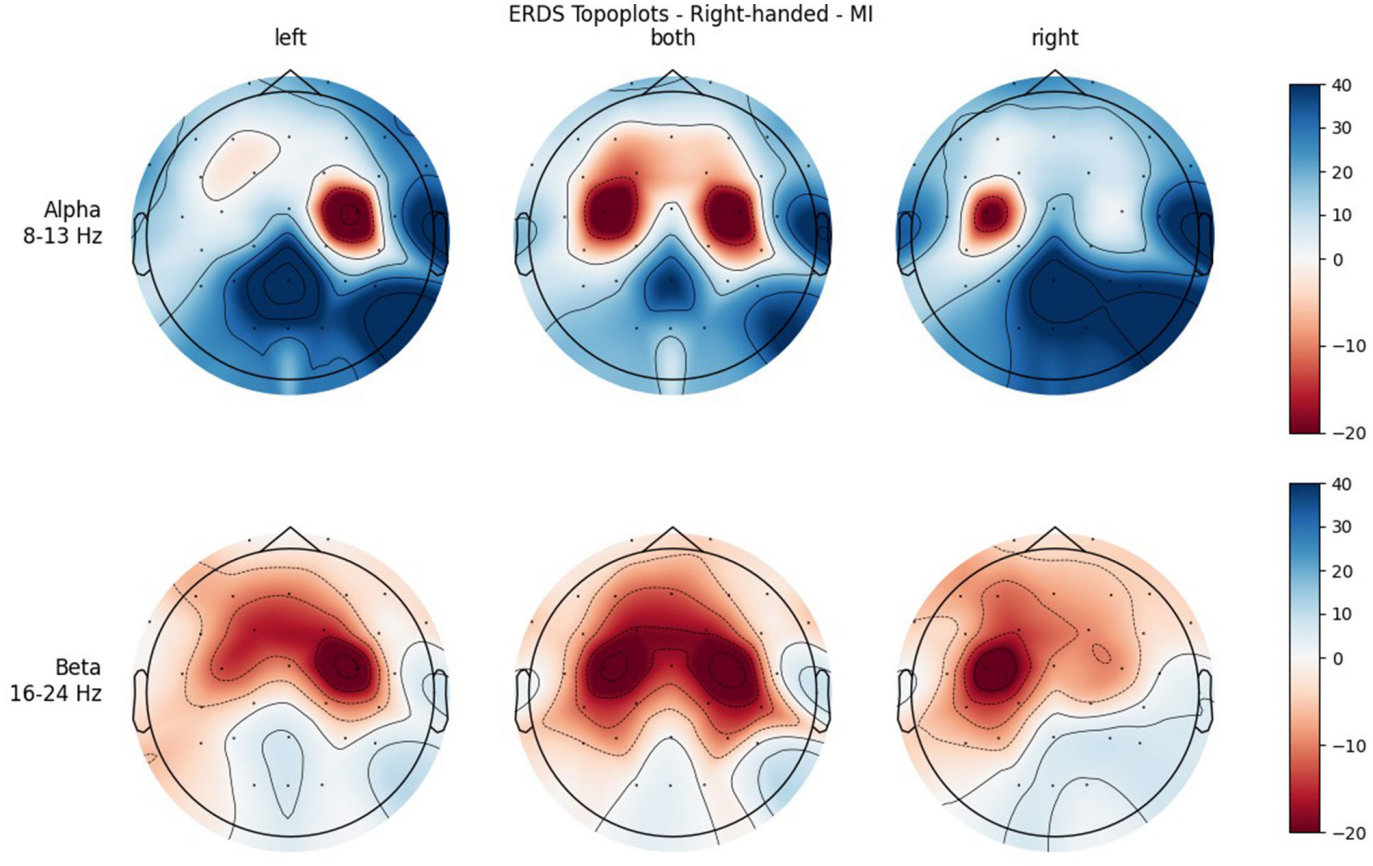
Topographical plots of mean ERD/S values (percentages relative to baseline interval) taken over all trials performed by right-handed participants during MI. Top row: alpha band (8–13 Hz); bottom row: beta band (16–24 Hz). Columns from left to right correspond to the three different conditions of left hand, both hands, and right hand movement. Red colors indicate ERD; blue colors, ERS.

Figures 4, 5 show the topoplots during ME of the left-handed and right-handed group, respectively. Figures 6, 7 analogously show the topoplots during MI. It can be seen that the strongest ERD can be found overall, around the electrodes C3 and C4. Occipital areas show the strongest ERS, especially in the alpha band.

The full tables of the statistical analysis are provided as Supplementary material.

#### 3.2.1. ME alpha band

The RMANOVA revealed a significant main effect for ROI [*F*_(3.53, 84.61)_ = 20.489, *p* < 0.001], indicating a significant difference between the six ROIs: FL (M = 10.212, SE = 3.76), FR (M = 10.085, SE = 3.44), CL (M = −0.881, SE = 3.27), CR (M = 1.911, SE = 3.29), PL (M = 9.330, SE = 2.98), and PR (M = 22.787, SE = 4.16). Furthermore, a significant interaction for Condition * Sex * Handedness was found [*F*_(1.72, 41.32)_ = 4.529, *p* = 0.021]; however, the paired-samples *post hoc* test revealed no statistical significance (smallest *p* = 0.264).

#### 3.2.2. ME beta band

Analysis for the beta band again showed a significant main effect for ROI [*F*_(3.02, 72.57)_ = 15.8790, *p* < 0.001], indicating a significant difference between the six ROIs: FL (M = −3.29, SE = 2.30), FR (M = −3.36, SE = 2.30), CL (M = −14.37, SE = 2.63), CR (M = −12.21, SE = 2.76), PL (M = −6.40, SE = 2.34), and PR (M = −2.83, SE = 2.55). Again, a significant interaction for Condition * Sex * Handedness was found [*F*_(1.68, 40.39)_ = 3.5916, *p* = 0.044], while the *post hoc* pairwise comparison did not show a statistical significance (smallest *p* = 0.454). Finally, a significant interaction for ROI * Condition was found [*F*_(7.03, 168.71)_ = 5.2472, *p* < 0.001].

#### 3.2.3. MI alpha band

As before, a significant main effect for ROI was found [*F*_(2.14, 51.36)_ = 20.2375, *p* < 0.001], indicating a significant difference between the six ROIs: FL (M = 4.05, SE = 2.98), FR (M = 5.75, SE = 2.99), CL (M = −6.24, SE = 3.37), CR (M = −6.12, SE = 3.75), PL (M = 10.69, SE = 3.37), and PR (M = 19.48, SE = 4.91). Another significant main effect was found for Condition [*F*_(1.82, 43.61)_ = 14.6404, *p* < 0.001], indicating a significant difference between the three conditions: BOTH (M = −2.48, SE = 3.34), LEFT (M = 7.21, SE = 3.23), and RIGHT (M = 9.07, SE = 3.30). Analysis also revealed a significant interaction for ROI * Condition [*F*_(5.00, 119.98)_ = 7.3841, *p* < 0.001].

#### 3.2.4. MI beta band

**Figure 8:**
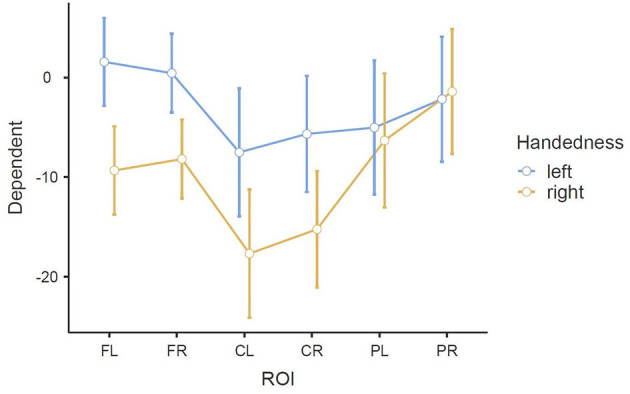
Estimated marginal means of the interaction ROI * Handedness in the beta band in the MI task (error bars: 95% confidence interval). y-axis: ERD/S values in percent (referenced to baseline interval).

As in all three RMANOVAs before, the main effect ROI reached significance [*F*_(3.01, 72.12)_ = 13.083, *p* < 0.001], indicating a significant difference between the six ROIs: FL (M = −3.88, SE = 1.51), FR (M = −3.88, SE = 1.36), CL (M = −12.60, SE = 2.20), CR (M = −10.45, SE = 2.00), PL (M = −5.68, SE = 2.30), and PR (M = −1.80, SE = 2.15). A significant difference was also found for the interaction of ROI * Handedness: *F*_(3.01, 72.12)_ = 4.635, *p* = 0.005. In the *post-hoc* test, only comparisons between the same ROIs (different handedness), or between the same handedness and opposite ROIs (i.e., FL-FR, CL-CR, PL-PR) were considered relevant to our research question. No such pair showed significance, although significance was almost reached for FL left—FL right: *t*_(24)_ = 3.598, *p* = 0.051. This difference can also be observed in the profile plot (Figure 8). Another significant main effect was found for Condition [*F*_(1.90, 45.65)_ = 6.374, *p* = 0.004], indicating a significant difference between the three conditions: BOTH (M = −8.77, SE = 1.73), LEFT (M = −5.84, SE = 1.63), and RIGHT (M = −4.53, SE = 1.97). A significant interaction was also found for ROI * Condition [*F*_(4.43, 106.26)_ = 4.274, *p* = 0.002]. Finally, concerning between-subjects effects, a tendency toward significance was found for the main effect Handedness: *F*_(1, 24)_ = 4.1035, *p* = 0.054, indicating a significant difference between the left-handedness (M = −3.06, SE = 2.32) and right-handedness (M = −9.70, SE = 2.32).

#### 3.2.5. Exemplary time-frequency maps

The ERD/S time-frequency maps of two exemplary participants for task MI are reported here. Both participants were of the same sex, the same handedness, and almost the same age. The rows of each figure represent the ROIs (Center Left and Center Right) within which the mean of all channels was taken, while the three different conditions (LEFT, BOTH, RIGHT) are split by the columns.

**Figure 9:**
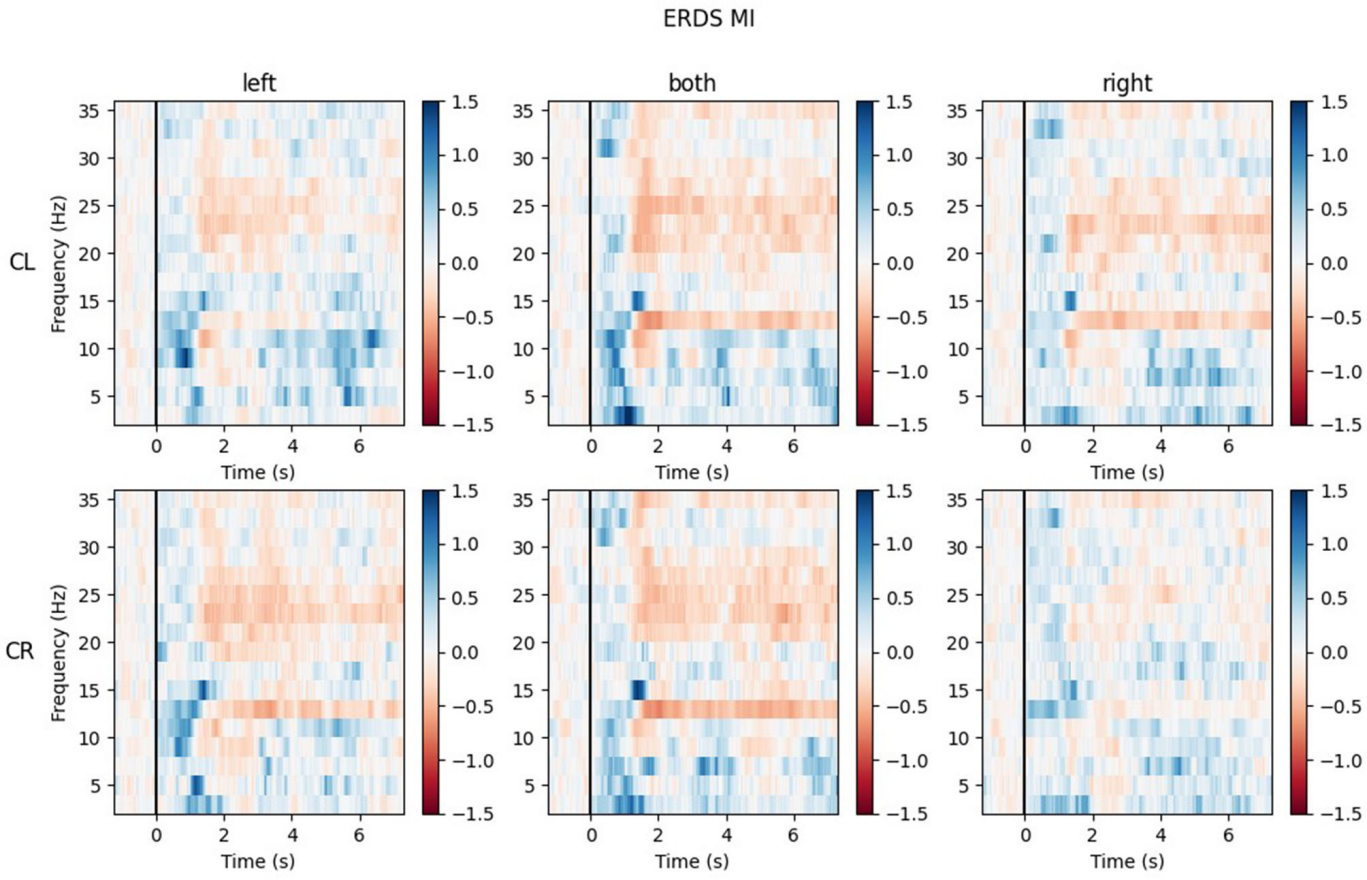
Time-frequency maps for participant P002 during MI for all conditions (columns from left to right: left hand, both hands, right hand movement) over both central ROIs (top row: central left ROI; bottom row: central right ROI). Red colors indicate ERD; blue colors, ERS.

Figure 9 shows the time-frequency maps of participant P002. ERD is strongly lateralized in the contralateral ROI for all conditions and especially in the alpha band quite narrow banded.

**Figure 10:**
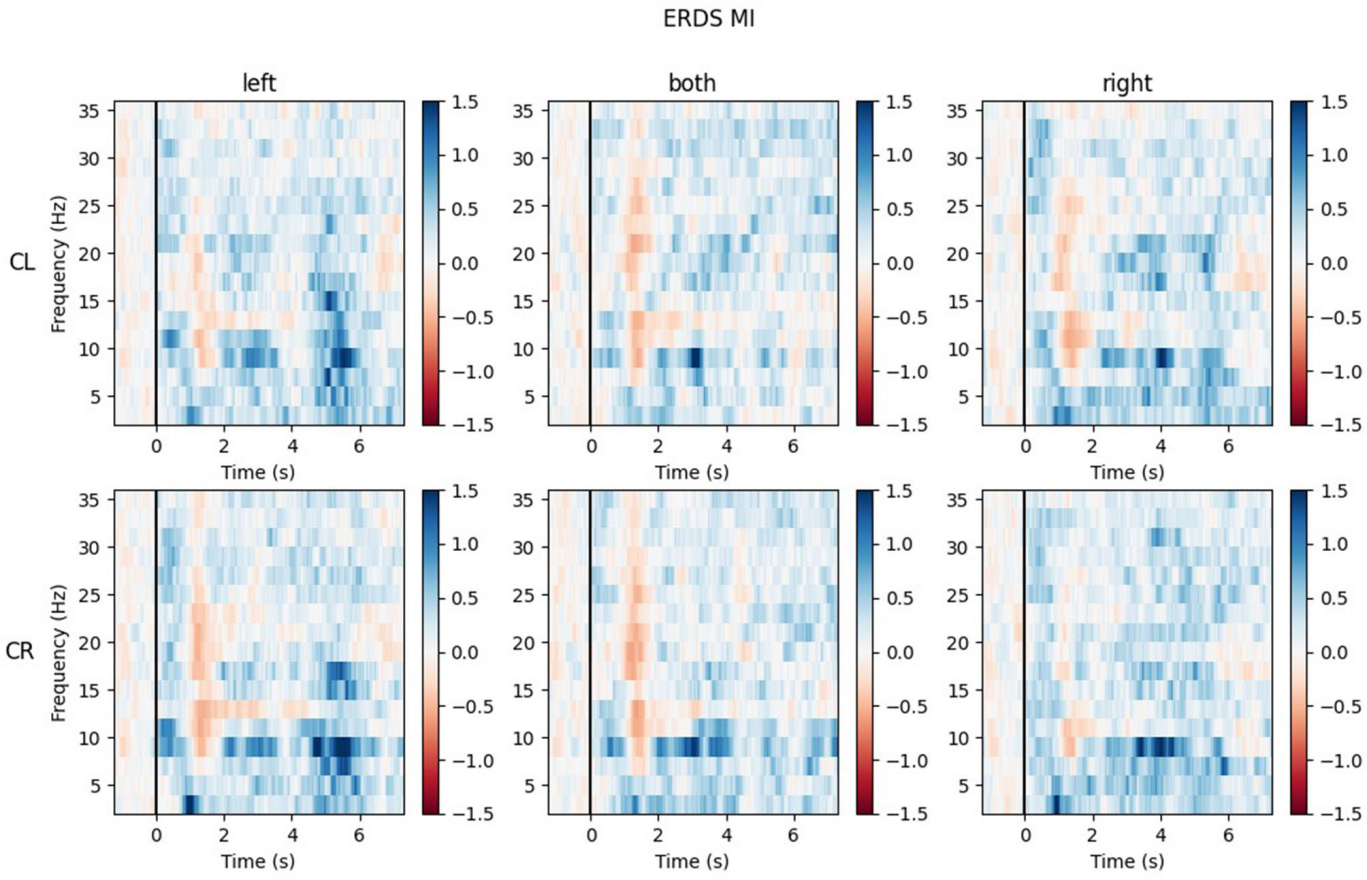
Time-frequency maps for participant P017 during MI for all conditions (columns from left to right: left hand, both hands, right hand movement) over both central ROIs (top row: central left ROI; bottom row: central right ROI). Red colors indicate ERD; blue colors, ERS.

Looking at the plots of participant P017 in Figure 10, the most apparent quality is that across all ROIs and conditions, there are barely any ERD patterns that remain over the whole action time interval. Most ERD activity can be found shortly after the cue onset (0.5–2 s) and in some maps again toward the end of the trial, during the last second. Rather than ERD, strong ERS patterns can be found in the lower alpha band (8–9 Hz) and delta band (1–4 Hz), especially during BOTH condition.

The maps for task ME (included in Supplementary material) show similar results, but will not be discussed further here.

## 4. Discussion

The present study investigated whether there are differences in the brain activation, specifically ERD/S patterns, during the imaging of simple hand movements that are caused by handedness. The sex of participants was also considered as a between-subject factor.

It is widely recognized that ME and MI rely on partly overlapping mechanisms. While the focus of this study was on the differences between brain activation of left- and right-handed people during MI, the differences during ME were also investigated.

The results of the VMIQ-2 questionnaire were similar for both handedness groups. From that, we conclude that differences found between these two groups during MI do not arise because of differences in the ability to perform MI.

### 4.1. Results of ME

A significant main effect for ROI was found in the statistical analysis for both frequency bands (alpha and beta), which was expected as it is already known that the most apparent activation patterns during movement execution and imagery can be found around the positions of the electrodes C3 and C4, reflecting the hand area (Pfurtscheller and Da Silva, 1999; Pfurtscheller et al., 2006).

Furthermore, when looking at the group ERD/S topoplots, it can be seen that in the alpha band, ERD is strongly localized around C3 and C4 only on the contralateral side. It should be noted that this lateralization appears clearer in the left-handed group. Previous studies found that left-handers showed more bilateral activation (Stancák Jr and Pfurtscheller, 1996; Solodkin et al., 2001; Pool et al., 2014; Crotti et al., 2022) and fewer functional asymmetries (Galaburda et al., 1978; Pool et al., 2014) than right-handers. As activation is tightly localized around C3 and C4, it can easily get lost when taking the average of the whole central ROI. Furthermore, considering the topoplots, the beta band shows much broader ERD patterns and weaker ERS as opposed to the alpha band.

The *post-hoc* analysis of the significant interaction Condition * Sex * Handedness revealed no significance in any frequency band. A look at the estimated marginal means of the alpha band reveals that the strongest ERS for left-handed men can be found in condition LEFT, while for right-handed men that is the condition where the least ERS is present. A similar pattern can also be recognized in the beta band, but with a shift toward a stronger ERS overall. Previous research also suggests that activation is stronger when the dominant hand is used as opposed to the non-dominant hand (Stancák Jr and Pfurtscheller, 1996; Crotti et al., 2022). Although it did not reach statistical significance, this finding is still worth mentioning. The significant interaction ROI * Condition was also to be expected (due to lateralization effects in the LEFT and RIGHT conditions), but was only found in the beta band, while in the alpha band only a weak tendency toward significance (*p* = 0.085) could be observed.

### 4.2. Results of MI

A significant main effect for ROI was found in both frequency bands, which was also expected.

In both bands, a strong ERD is located in the central ROIs, which contain the channels C3 and C4. In the alpha band, the other ROIs show ERS, mostly in the parietal right ROI, while in the beta band the ERD also extends to those regions. Similar to ME, activation is more localized in the alpha band, while in the beta band broad bilateral activation patterns can be found.

The significant interaction ROI * Handedness was only found in the beta band. *Post-hoc*, we looked for significance in pairings of the same ROIs but different handedness, and also pairings of the same-handedness and opposite (left vs. right side) ROIs. No such significant pairs were found, but there was a tendency toward significance for the pairing FL left–FL right. The marginal means indicate that differences between the two-handedness groups exist only in the frontal and central ROIs, where the left-handed group shows less ERD, but not in parietal ROIs. Parieto-occipital regions might show the least differences because these are where there is strong ERS in all the participants.

A significant main effect was also found for Condition in both frequency bands, with the mean value of BOTH condition reflecting stronger ERD than the mean values of the unimanual conditions. This can be attributed to the fact that BOTH condition recruits bilateral networks, while the other conditions show more lateralized patterns (see topoplots), which is in line with the literature (Walsh et al., 2008). In the alpha band, where ERD is especially localized, the mean values for the LEFT and RIGHT condition are positive, meaning that averaged over all ROIs there is more ERS than ERD. Even in BOTH condition the mean value is only slightly negative while there is a relatively large standard error, which cannot be said to reflect ERD. However, in the beta band there are much broader ERD patterns, which is apparent in both the topoplots and the mean values of the conditions, which are clearly negative.

Another significant interaction was found for ROI * Condition in both frequency bands. While there is basically no difference between the central ROIs in BOTH condition, there is in the LEFT and RIGHT conditions, and they are reversed: In RIGHT condition, there is ERD in the left and ERS in the right ROI, whereas in condition LEFT there is ERD in the right and ERS in the left ROI. This is interpreted as contralateral activation and ipsilateral deactivation, which is in line with an early study by Pfurtscheller et al. (1997) and also confirms what we hypothesized. The lateralization effect is more pronounced in the alpha than in the beta band.

It was only in the beta band that handedness showed a tendency toward significance, with the right-handed group having a more negative mean ERD/S value than left-handers. Looking at the topoplots, it can be seen that in right-handers there is strong and very broad bilateral ERD, while only weak ERS. In the left-handed group, ERD is less broad, a bit more lateralized, and overall weaker. Simultaneously, this group shows more ERS than their right-handed counterpart, especially in the LEFT and RIGHT conditions. A similar result can be observed in the alpha band, although to a lesser extent, which is why no (tendency toward) significance could be found in that frequency band. This finding is in contrast with our hypothesis, in which we expected more bilateral ERD in left-handed participants than in right-handed participants. This is quite surprising, as research so far has suggested that brain activation and effective connectivity are less asymmetric in the motor system of left-handed individuals (Solodkin et al., 2001; Willems et al., 2009; Pool et al., 2014; Zapała et al., 2020).

### 4.3. Inter-subject variability

We included the grand average ERD/S time-frequency maps of the handedness groups as Supplementary material only, because these added no value to our research question. The inter-subject variability is so pronounced that taking the mean over many participants cancels out the individual patterns. We specifically reported the results of two exemplary individuals to demonstrate how different the brain patterns of two participants can be, even when they are of the same handedness and sex. Wriessnegger et al. (2020) recently performed an analysis of (dis)similarities on the data of one of their previous studies observing great variability. They confirmed high interindividual differences during MI, especially in the alpha band. This high variability between participants can be a problem when taking the mean over groups. Future studies should take the variation of (dis)similarities into account.

### 4.4. Limitations

Our study labeled participants as either left- or right-handed, without considering the degree of handedness. Looking at more participants and considering their degree of handedness, for example, represented by their EHI laterality index or by grouping them into narrower defined subgroups of handedness might lead to more fine-grained results and ultimately to finding a possible continuous relationship between handedness and differences in brain activation. Furthermore, this study did not consider people who were born as left-handers but later learned (either voluntarily or involuntarily) to mainly use their right hand. The influences of this “retraining” on brain patterns could be a research topic on its own. A meta-analysis (Papadatou-Pastou et al., 2020) suggests that the prevalence of mixed-handedness is 9.33%—a rate almost as high as for left-handedness, highlighting the need of including this group in future analyses.

Furthermore, in this study, only kinesthetic motor imagery was used. In a study conducted by Zapała et al. (2021), left-handed participants obtained higher power in the alpha band during visual MI as opposed to kinesthetic MI. The meta-analysis by Hétu et al. (2013) also concluded that the MI modality could influence the consistency of brain activation. Thus, the question arises of whether the same results were to have been expected if we had used a different MI strategy in our study.

## 5. Conclusion

We investigated the EEG correlates of both left- and right-handed people during ME and MI of a simple repetitive hand movement using either the dominant, non-dominant, or both hands, all in one study. This provides a complement to a recent study by Crotti et al. (2022) in which a similar experiment was conducted using fMRI instead of EEG.

Our main hypothesis—that left-handed participants will show more bilateral activation than right-handed participants—could not be confirmed. In fact, in the alpha band during ME and in both the alpha and the beta band during MI, we observed the opposite. The other hypotheses could (partially) be confirmed. The strongest ERD was found over sensorimotor areas. The ERD was strongest in the contralateral hemisphere of the hand used in most cases. One example counter to this was demonstrated by the left-handed group during ME, which always showed the strongest ERD in the left hemisphere, regardless of the hand used. In general, similar activation patterns were raised by ME and MI, although they were richer in contrast during MI than during ME.

This study provides another step in the process of identifying and understanding the differences between left- and right-handed people on the neural level. Our findings could for example lead to an improved BCI performance especially for left-handed users, as so far most experiments only focused on right-handed people. While we investigated ERD/S patterns, a deeper look at the differences in brain connectivity can be taken in future.

## Data availability statement

The datasets presented in this study can be found in online repositories. The names of the repository/repositories and accession number(s) can be found below: Raw EEG data of Lajtos et al. (2023) Effects of handedness on brain oscillatory activity during imagery and execution of upper limb movements https://osf.io/ntpyb/?view_only=2fc19c4ba8a84a2ab8aaec7a8f881dbe.

## Ethics statement

The studies involving human participants were reviewed and approved by Medical University Graz. The patients/participants provided their written informed consent to participate in this study.

## Author contributions

ML, LB-C, and SW conceived and designed the experiments, wrote, reviewed, and edited the manuscript. ML performed the experiments and wrote the original draft of the manuscript. ML and LB-C analyzed the data. SW supervised the study. All authors contributed to the article and approved the submitted version.
