# Supplementary material for: Effects of handedness on brain oscillatory activity during imagery and execution of upper limb movements
Source: Front Psychol. 2023 Jun 13;14:1161613. doi: 10.3389/fpsyg.2023.1161613 (PMC10293623; doi:10.3389/fpsyg.2023.1161613)
Supplement: Supplementary file 1 [file Data_Sheet_1.PDF]

Supplementary Material

1 ADDITIONAL DISCUSSION

1.1 Comparison ME vs. MI

Since no statistical tests were performed with ME and MI as factors, the following findings were only drawn from the group ERD/S topoplots: In the right-handed group, it was found that activation in the alpha band is stronger in amplitude and more lateralized during MI compared to ME. In the beta band, the activation patterns appear more similar. This is in contrast with our previous study which revealed more bilateral activation in (only) right-handers during MI compared to during ME (Crotti et al., 2022). The opposite was found for the left-handed group. Here, activation patterns of ME and MI look mostly similar in the alpha band, with just slightly more lateralization and some ERS in the ipsilateral hemisphere during (unimanual) MI. In the beta band, activation is much stronger during MI. For both ME and MI we found that activation is more localized (mostly in the contralateral hemisphere) in the alpha band. The beta band shows broader and bilateral activation patterns in all conditions and both handedness groups.

The significant interaction ROI * Condition was only found in the beta band in ME, but in both bands for MI. It was already mentioned that in the right-handed group the expected contralateral activation could not be found in the alpha band. Specifically, the ERD/S patterns of the LEFT and BOTH conditions look alike, with both showing bilateral activation. What also might be a reason for not reaching significance is that we looked at ROIs instead of just the C3 and C4 channels. Because ERD is very localized in the alpha band and surrounded by ERS, these effects cancel each other out when taking the average over the central ROIs. Thus, ERD needs to be very strong in amplitude and/or broader in order to "survive" the averaging, as was the case for the alpha band in MI.

Similarly, the main effect Condition reached significance only for MI, but not for ME. This has different reasons in the alpha and the beta band. In the alpha band, as just mentioned, ERD is just too weak during ME and, especially in the right-handed group, lacks lateralization based on Condition. While there is a bilateral activation for both ME and MI in the beta band, the strongest ERD can still be clearly seen to be in the contralateral hemisphere in the MI task, whereas during ME the left-handed group always showed the strongest ERD in the left hemisphere, regardless of the Condition.

To sum up, one can say that at large, the participants of this study showed similar activation patterns while performing ME and MI, which is in line with our hypothesis and with what Crotti et al. (Crotti et al., 2022) found in their fMRI study. However, some differences could be found, the most apparent one being that activation patterns are richer in contrast (i.e. presenting higher amplitudes for both ERS and ERD) during MI than during ME. An explanation for this finding might be that while performing MI the participants were more focused on the task. Since the hand movement used for this study (repetitively squeezing a ball) represents a rather simple task, participants might not have been consciously engaged in it during ME. Many participants reported difficulties performing the MI task and that they had to put in a lot of effort especially during the first few trials and in trials of only their non-dominant hand.

1.2 Sex Differences

The RMANOVAs for both alpha and beta band during ME showed a significant interaction for Condition * Sex * Handedness, however, no statistical significance was revealed in post-hoc analysis. Looking at the

estimated marginal means showed some slight differences between the sexes which have already been discussed. We saw that males might tend to display more distinct activation patterns between different conditions than females.

2 ADDITIONAL TIME-FREQUENCY MAPS

2.1 Grand Averages of Handedness Groups

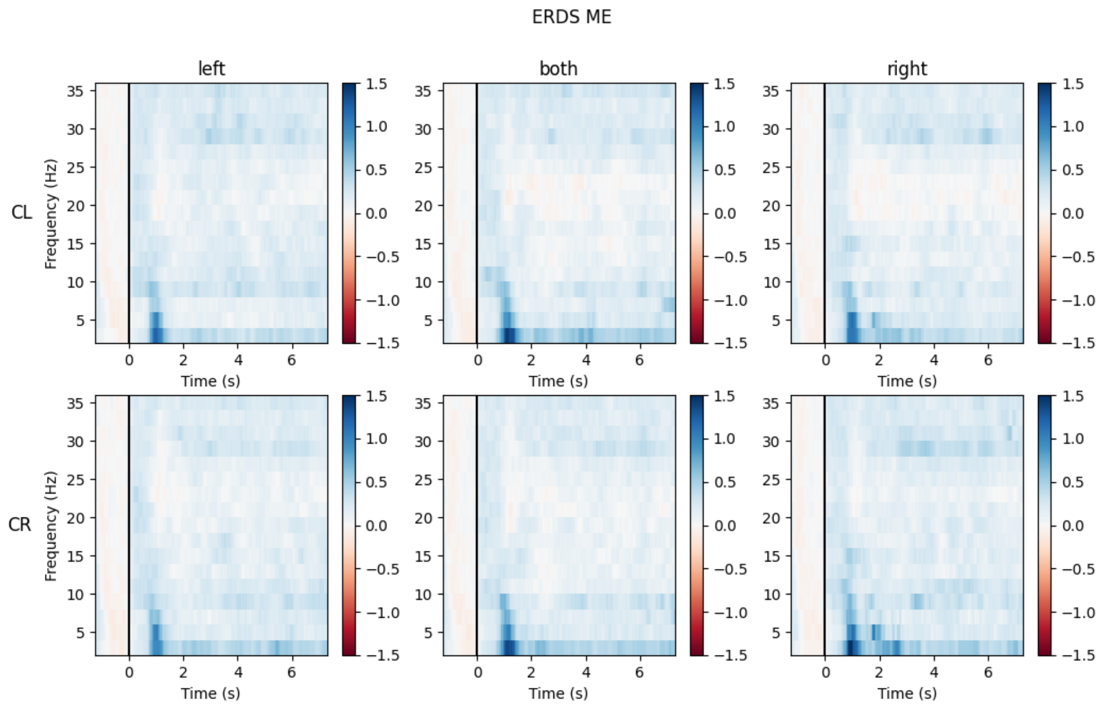

Figure S1. Grand average time-frequency maps for all left-handed participants during ME for all conditions (columns from left to right: left hand, both hands, right hand movement) over both central ROIs (top row: central left ROI; bottom row: central right ROI). Red colors indicate ERD, blue colors ERS.

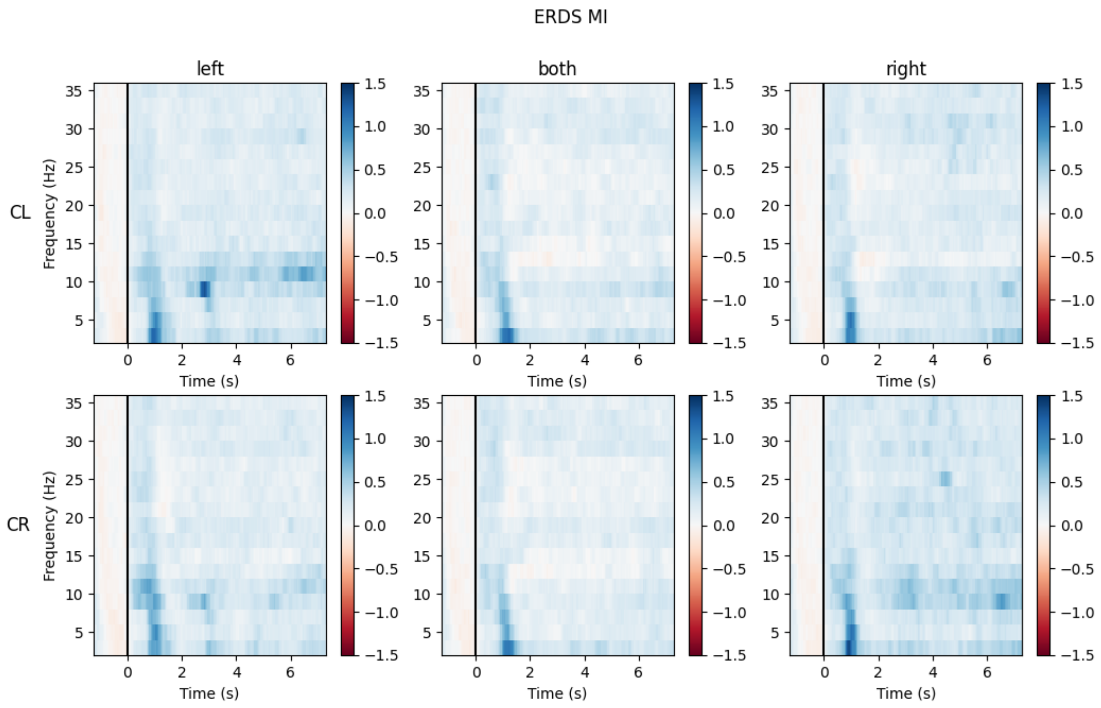

Figure S2. Grand average time-frequency maps for all left-handed participants during MI for all conditions (columns from left to right: left hand, both hands, right hand movement) over both central ROIs (top row: central left ROI; bottom row: central right ROI). Red colors indicate ERD, blue colors ERS.

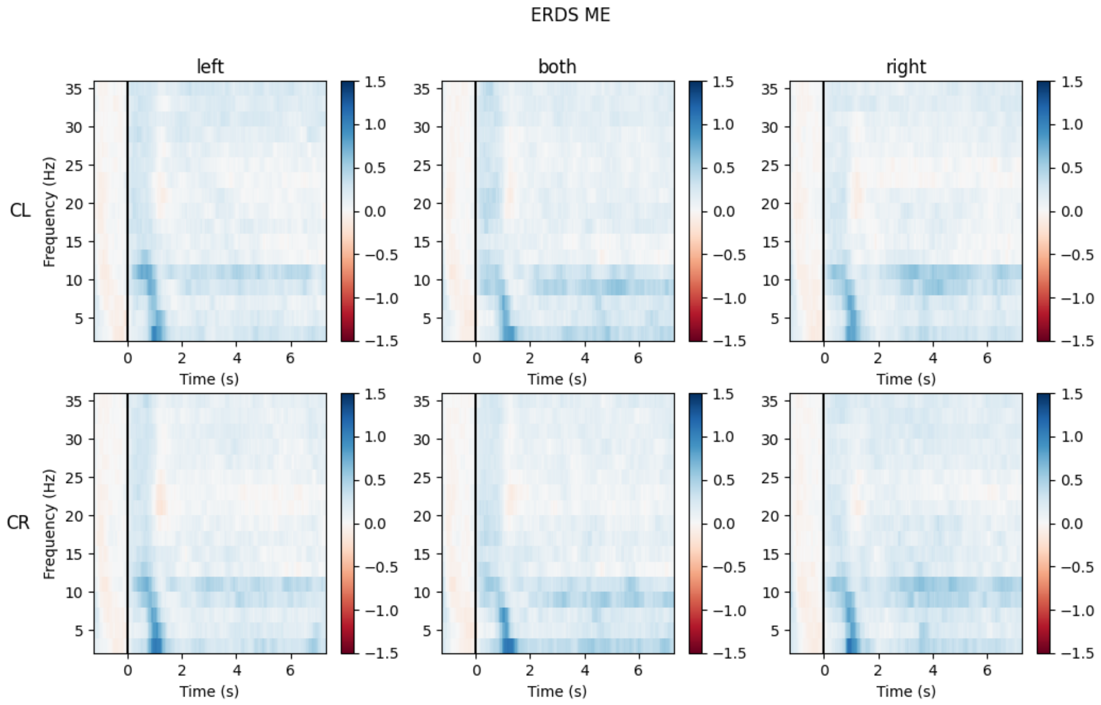

Figure S3. Grand average time-frequency maps for all right-handed participants during ME for all conditions (columns from left to right: left hand, both hands, right hand movement) over both central ROIs (top row: central left ROI; bottom row: central right ROI). Red colors indicate ERD, blue colors ERS.

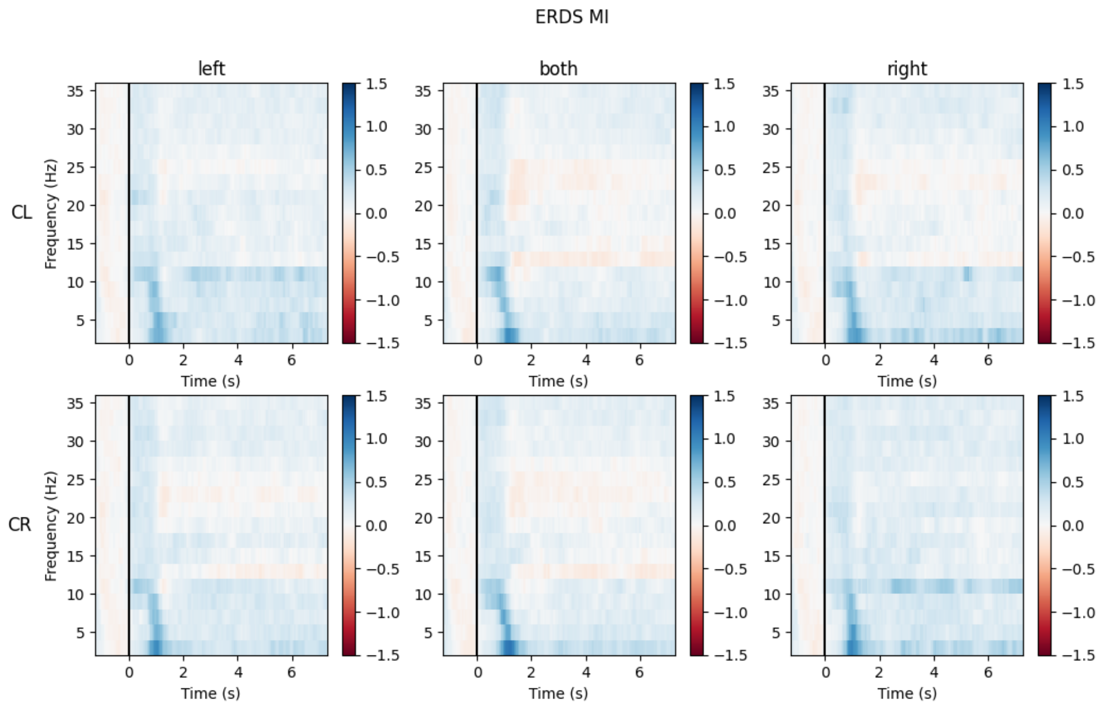

Figure S4. Grand average time-frequency maps for all right-handed participants during MI for all conditions (columns from left to right: left hand, both hands, right hand movement) over both central ROIs (top row: central left ROI; bottom row: central right ROI). Red colors indicate ERD, blue colors ERS.

2.2 Exemplary Participants

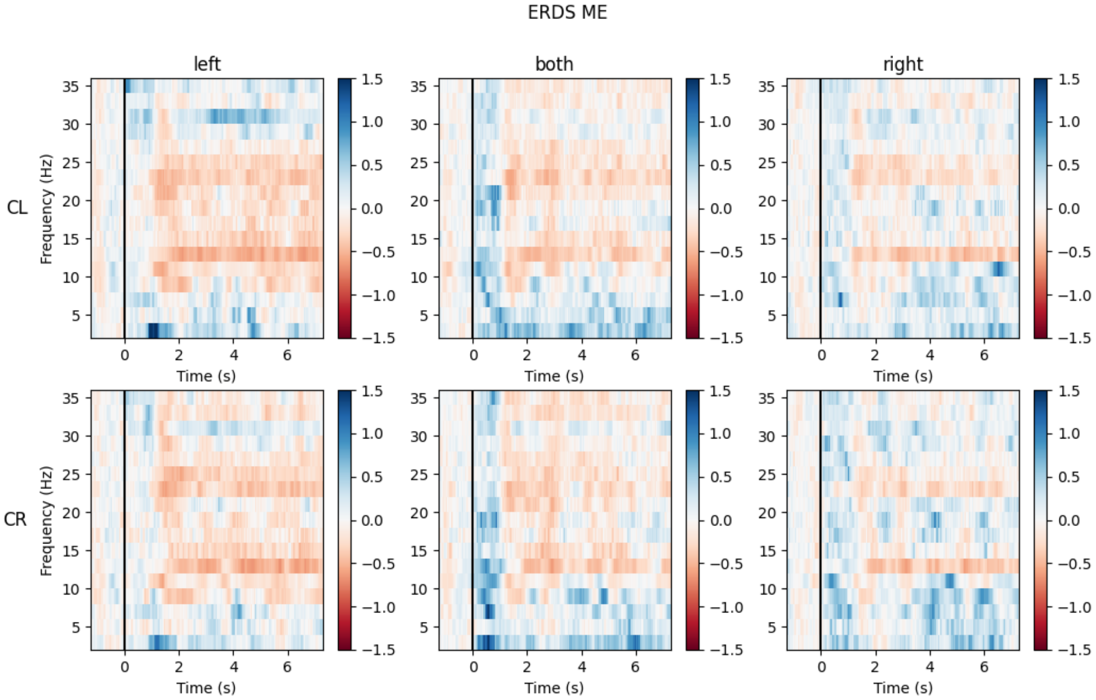

Figure S5. Time-frequency maps for participant P002 during ME for all conditions (columns from left to right: left hand, both hands, right hand movement) over both central ROIs (top row: central left ROI; bottom row: central right ROI). Red colors indicate ERD, blue colors ERS.

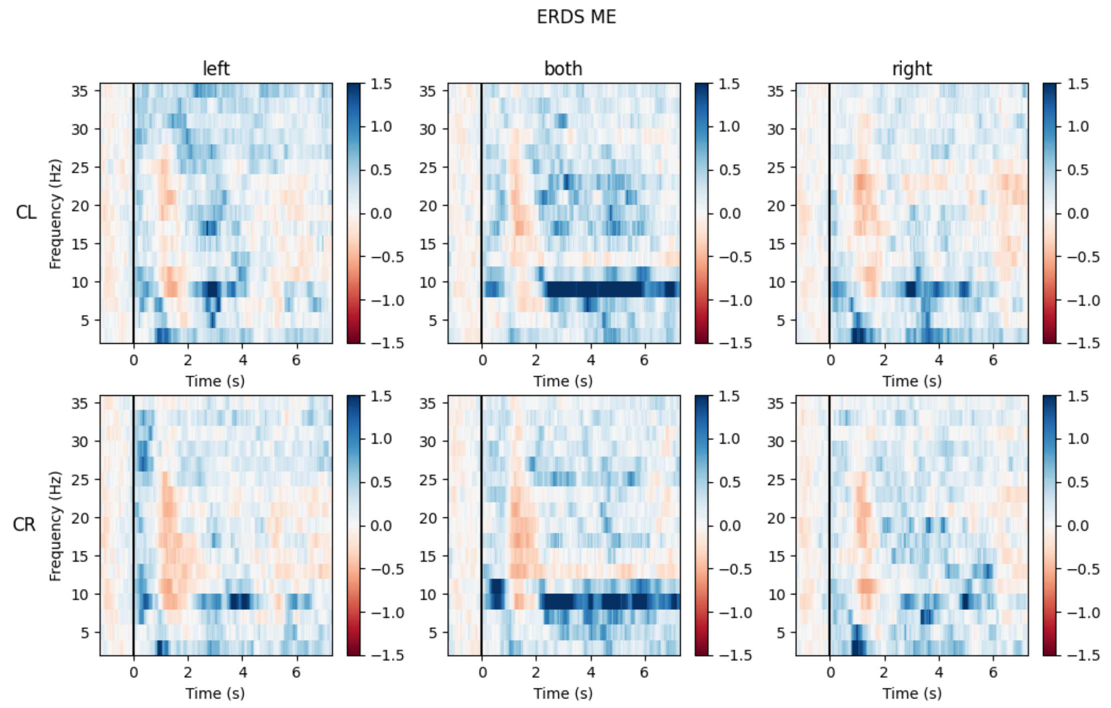

Figure S6. Time-frequency maps for participant P017 during ME for all conditions (columns from left to right: left hand, both hands, right hand movement) over both central ROIs (top row: central left ROI; bottom row: central right ROI). Red colors indicate ERD, blue colors ERS.

3 REPEATED MEASURES ANOVA TABLES

Within Subjects Effects						
	**Sphericity Correction**	**df**	**Mean Square**	**F**	**p**	
ROI	None	5	5723.1	20.489	< .001	**
	Greenhouse-Geisser	3.53	8117.1	20.489	< .001	**
ROI ∗ Sex	None	5	338.8	1.213	0.307	
	Greenhouse-Geisser	3.53	480.6	1.213	0.311	
ROI ∗ Handedness	None	5	219.1	0.784	0.563	
	Greenhouse-Geisser	3.53	310.8	0.784	0.524	
ROI ∗ Sex ∗ Handedness	None	5	136.9	0.490	0.783	
	Greenhouse-Geisser	3.53	194.2	0.490	0.720	
Residual	None	120	279.3			
	Greenhouse-Geisser	84.61	396.2			
Condition	None	2	1275.4	1.716	0.191	
	Greenhouse-Geisser	1.72	1481.7	1.716	0.195	
Condition ∗ Sex	None	2	79.9	0.107	0.898	
	Greenhouse-Geisser	1.72	92.8	0.107	0.870	
Condition ∗ Handedness	None	2	1348.3	1.814	0.174	
	Greenhouse-Geisser	1.72	1566.4	1.814	0.180	
Condition ∗ Sex ∗ Handedness	None	2	3365.9	4.529	0.016	*
	Greenhouse-Geisser	1.72	3910.4	4.529	0.021	*
Residual	None	48	743.2			
	Greenhouse-Geisser	41.32	863.4			
ROI ∗ Condition	None	10	156.6	1.912	0.044	*
	Greenhouse-Geisser	5.80	269.9	1.912	0.085	
ROI ∗ Condition ∗ Sex	None	10	151.0	1.843	0.054	
	Greenhouse-Geisser	5.80	260.2	1.843	0.097	
ROI ∗ Condition ∗ Handedness	None	10	58.8	0.718	0.707	
	Greenhouse-Geisser	5.80	101.3	0.718	0.631	
ROI ∗ Condition ∗	None	10	50.5	0.616	0.799	
	Greenhouse-Geisser	5.80	87.0	0.616	0.711	
Residual	None	240	81.9			
	Greenhouse-Geisser	139.28	141.2			

Table S1. RMANOVA ME Alpha band - Within subjects effects.

Between Subjects Effects				
	**df**	**Mean Square**	**F**	**p**
Sex	1	412	0.0860	0.772
Handedness	1	12165	2.5400	0.124
Sex ∗ Handedness	1	2092	0.4367	0.515
Residual	24	4789		

Table S2. RMANOVA for Motor Execution Alpha band - Between subjects effects.

Tests of Sphericity					
	**Mauchly's W**	**p**	**Greenhouse-Geisser** ϵ	**Huynh-Feldt** ϵ	
ROI	0.1989	0.001	0.705	0.841	*
Condition	0.8382	0.131	0.861	0.921	
ROI ∗ Condition	0.0101	< .001	0.580	0.786	**

Table S3. RMANOVA for Motor Execution Alpha band - Tests of sphericity.

Within Subjects Effects						
	**Sphericity Correction**	**df**	**Mean Square**	**F**	**p**	
ROI	None	5	2119.3	15.8790	< .001	**
	Greenhouse-Geisser	3.02	3504.4	15.8790	< .001	**
ROI ∗ Sex	None	5	49.0	0.3671	0.870	
	Greenhouse-Geisser	3.02	81.0	0.3671	0.779	
ROI ∗ Handedness	None	5	106.3	0.7965	0.554	
	Greenhouse-Geisser	3.02	175.8	0.7965	0.501	
ROI ∗ Sex ∗ Handedness	None	5	21.0	0.1577	0.977	
	Greenhouse-Geisser	3.02	34.8	0.1577	0.926	
Residual	None	120	133.5			
	Greenhouse-Geisser	72.57	220.7			
Condition	None	2	91.4	0.5976	0.554	
	Greenhouse-Geisser	1.68	108.6	0.5976	0.527	
Condition ∗ Sex	None	2	12.8	0.0836	0.920	
	Greenhouse-Geisser	1.68	15.2	0.0836	0.890	
Condition ∗ Handedness	None	2	129.9	0.8495	0.434	
	Greenhouse-Geisser	1.68	154.4	0.8495	0.418	
Condition ∗ Sex ∗ Handedness	None	2	549.2	3.5916	0.035	*
	Greenhouse-Geisser	1.68	652.7	3.5916	0.044	*
Residual	None	48	152.9			
	Greenhouse-Geisser	40.39	181.7			
ROI ∗ Condition	None	10	111.2	5.2472	< .001	**
	Greenhouse-Geisser	7.03	158.1	5.2472	< .001	**
ROI ∗ Condition ∗ Sex	None	10	14.5	0.6831	0.740	
	Greenhouse-Geisser	7.03	20.6	0.6831	0.687	
ROI ∗ Condition ∗ Handedness	None	10	15.0	0.7082	0.716	
	Greenhouse-Geisser	7.03	21.3	0.7082	0.666	
ROI ∗ Condition ∗ Sex ∗ Handedness	None	10	21.5	1.0130	0.433	
	Greenhouse-Geisser	7.03	30.5	1.0130	0.424	
Residual	None	240	21.2			
	Greenhouse-Geisser	168.71	30.1			

Table S4. RMANOVA for Motor Execution Beta band - Within subject effects.

Between Subjects Effects				
	**df**	**Mean Square**	**F**	**p**
Sex	1	13.6	0.00557	0.941
Handedness	1	463.5	0.18954	0.667
Sex ∗ Handedness	1	644.9	0.26375	0.612
Residual	24	2445.3		

Table S5. RMANOVA for Motor Execution Beta band - Between subject effects.

Tests of Sphericity					
	**Mauchly's W**	**p**	**Greenhouse-Geisser ϵ**	**Huynh-Feldt ϵ**	
ROI	0.150	< .001	0.605	0.702	**
Condition	0.812	0.091	0.841	0.898	
ROI ∗ Condition	0.105	0.790	0.703	1.000	

Table S6. RMANOVA for Motor Execution Beta band - Tests of sphericity.

Post Hoc Comparisons - ROI ∗ Condition									
**Comparison**									
**ROI**	**Condition**		**ROI**	**Condition**	**Mean Difference**	**SE**	**df**	**t**	$\mathbf{p}_{tukey}$
FL	Both	-	CL	Both	9.6043	1.72	24.0	5.5721	0.001
		-	CL	Left	9.4411	1.92	24.0	4.9075	0.005
		-	CL	Right	10.3539	2.25	24.0	4.6115	0.010
		-	CR	Both	8.7775	1.71	24.0	5.1341	0.003
		-	CR	Left	9.3064	2.29	24.0	4.0623	0.034
	Left	-	CL	Both	12.5236	2.16	24.0	5.7904	< .001
		-	CL	Left	12.3604	1.90	24.0	6.4893	< .001
		-	CL	Right	13.2732	2.10	24.0	6.3261	< .001
		-	CR	Both	11.6968	2.20	24.0	5.3177	0.002
		-	CR	Left	12.2257	2.06	24.0	5.9267	< .001
	Right	-	CL	Both	10.5343	2.33	24.0	4.5179	0.012
		-	CL	Left	10.3711	1.98	24.0	5.2491	0.002
		-	CL	Right	11.2839	1.77	24.0	6.3709	< .001
		-	CR	Both	9.7075	2.38	24.0	4.0720	0.033
		-	CR	Left	10.2364	2.38	24.0	4.2935	0.020
FR	Both	-	CL	Both	11.4818	1.73	24.0	6.6491	< .001
		-	CL	Left	11.3186	2.11	24.0	5.3575	0.002
		-	CL	Right	12.2314	2.38	24.0	5.1447	0.003
		-	CR	Both	10.6550	1.62	24.0	6.5591	< .001
		-	CR	Left	11.1839	2.21	24.0	5.0698	0.003
	Left	-	CL	Both	9.7550	2.40	24.0	4.0595	0.034
		-	CL	Left	9.5918	2.10	24.0	4.5602	0.011
		-	CL	Right	10.5046	2.18	24.0	4.8246	0.006
		-	CR	Left	9.4571	2.09	24.0	4.5194	0.012
	Right	-	CL	Both	11.1971	2.36	24.0	4.7418	0.007
		-	CL	Left	11.0339	2.20	24.0	5.0178	0.004
		-	CL	Right	11.9468	1.86	24.0	6.4084	< .001
		-	CR	Both	10.3704	2.26	24.0	4.5820	0.010
		-	CR	Left	10.8993	2.23	24.0	4.8895	0.005
		-	CR	Right	6.4311	1.66	24.0	3.8851	0.050
CL	Both	-	PL	Both	-8.6136	2.02	24.0	-4.2742	0.021
		-	PR	Both	-9.5682	2.22	24.0	-4.3117	0.019
		-	PR	Right	-15.4261	3.08	24.0	-5.0067	0.004
	Left	-	PL	Both	-8.4504	1.97	24.0	-4.2959	0.020
		-	PR	Both	-9.4050	2.03	24.0	-4.6248	0.010
		-	PR	Right	-15.2629	2.66	24.0	-5.7313	< .001
	Right	-	PR	Both	-10.3179	2.67	24.0	-3.8577	0.053
		-	PR	Right	-16.1757	2.83	24.0	-5.7146	< .001
CR	Both	-	PL	Both	-7.7868	1.94	24.0	-4.0114	0.038
		-	PR	Both	-8.7414	2.13	24.0	-4.1135	0.030
		-	PR	Right	-14.5993	3.23	24.0	-4.5205	0.012
	Left	-	PL	Both	-8.3157	2.14	24.0	-3.8860	0.050
		-	PR	Both	-9.2704	2.04	24.0	-4.5435	0.011
		-	PR	Left	-8.7261	2.06	24.0	-4.2286	0.024
		-	PR	Right	-15.1282	2.78	24.0	-5.4372	0.001
	Right	-	PR	Right	-10.6600	2.57	24.0	-4.1410	0.029

Table S7. RMANOVA for Motor Execution Beta band - Post Hoc Comparisons - ROI ∗ Condition. Only statistically significant comparisons are shown.

Within Subjects Effects						
	**Sphericity Correction**	**df**	**Mean Square**	**F**	**p**	
ROI	None	5	8275.1	20.2375	< .001	**
	Greenhouse-Geisser	2.14	19334.7	20.2375	< .001	**
ROI ∗ Sex	None	5	384.8	0.9410	0.457	
	Greenhouse-Geisser	2.14	899.0	0.9410	0.402	
ROI ∗ Handedness	None	5	900.7	2.2027	0.058	
	Greenhouse-Geisser	2.14	2104.4	2.2027	0.118	
ROI ∗ Sex ∗ Handedness	None	5	282.4	0.6905	0.632	
	Greenhouse-Geisser	2.14	659.7	0.6905	0.515	
Residual	None	120	408.9			
	Greenhouse-Geisser	51.36	955.4			
Condition	None	2	6467.9	14.6404	< .001	**
	Greenhouse-Geisser	1.82	7118.6	14.6404	< .001	**
Condition ∗ Sex	None	2	16.8	0.0380	0.963	
	Greenhouse-Geisser	1.82	18.5	0.0380	0.952	
Condition ∗ Handedness	None	2	12.0	0.0273	0.973	
	Greenhouse-Geisser	1.82	13.3	0.0273	0.965	
Condition ∗ Sex ∗ Handedness	None	2	1167.1	2.6419	0.082	
	Greenhouse-Geisser	1.82	1284.6	2.6419	0.087	
Residual	None	48	441.8			
	Greenhouse-Geisser	43.61	486.2			
ROI ∗ Condition	None	10	718.1	7.3841	< .001	**
	Greenhouse-Geisser	5.00	1436.6	7.3841	< .001	**
ROI ∗ Condition ∗ Sex	None	10	94.1	0.9677	0.472	
	Greenhouse-Geisser	5.00	188.3	0.9677	0.440	
ROI ∗ Condition ∗ Handedness	None	10	72.3	0.7434	0.683	
	Greenhouse-Geisser	5.00	144.6	0.7434	0.592	
ROI ∗ Condition ∗ Sex ∗ Handedness	None	10	123.5	1.2703	0.248	
	Greenhouse-Geisser	5.00	247.1	1.2703	0.281	
Residual	None	240	97.3			
	Greenhouse-Geisser	119.98	194.5			

Table S8. RMANOVA for Motor Imagery Alpha band - Within subjects effects.

Between Subjects Effects				
	**df**	**Mean Square**	**F**	**p**
Sex	1	102	0.0223	0.883
Handedness	1	908	0.1988	0.660
Sex ∗ Handedness	1	1490	0.3262	0.573
Residual	24	4567		

Table S9. RMANOVA for Motor Imagery Alpha band - Between subjects effects.

Tests of Sphericity					
	**Mauchly's W**	**p**	**Greenhouse-Geisser** ϵ	**Huynh-Feldt** ϵ	
ROI	0.0446	< .001	0.428	0.471	**
Condition	0.8994	0.295	0.909	0.979	
ROI ∗ Condition	0.0104	< .001	0.500	0.647	**

Table S10. RMANOVA for Motor Imagery Alpha band - Test of sphericity.

Post Hoc Comparisons - ROI ∗ Condition

Comparison									
**ROI**	**Condition**		**ROI**	**Condition**	**Mean Difference**	**SE**	**df**	**t**	**p_{tukey}**
FL	Both	-	CL	Both	14.8307	2.66	24.0	5.5672	0.001
		-	CR	Both	14.9093	3.03	24.0	4.9275	0.005
		-	PL	Left	-16.6796	3.69	24.0	-4.5240	0.012
		-	PR	Left	-19.8629	4.48	24.0	-4.4307	0.015
		-	PR	Right	-28.2675	5.52	24.0	-5.1211	0.003
	Left	-	CL	Both	19.0911	3.46	24.0	5.5146	0.001
		-	CR	Both	19.1696	3.61	24.0	5.3093	0.002
		-	CR	Left	14.3307	3.57	24.0	4.0150	0.038
		-	PR	Right	-24.0071	5.35	24.0	-4.4887	0.013
	Right	-	CL	Both	22.0736	3.62	24.0	6.0958	< .001
		-	CL	Right	14.2729	3.09	24.0	4.6137	0.010
		-	CR	Both	22.1521	3.81	24.0	5.8085	< .001
		-	CR	Left	17.3132	4.12	24.0	4.2069	0.025
		-	PR	Right	-21.0246	4.77	24.0	-4.4113	0.016
FR	Both	-	CL	Both	15.5121	2.90	24.0	5.3475	0.002
		-	CR	Both	15.5907	3.37	24.0	4.6286	0.009
		-	PL	Left	-15.9982	3.36	24.0	-4.7615	0.007
		-	PL	Right	-10.8461	2.68	24.0	-4.0415	0.036
		-	PR	Left	-19.1814	4.67	24.0	-4.1112	0.031
		-	PR	Right	-27.5861	4.86	24.0	-5.6711	< .001
	Left	-	CL	Both	23.5943	3.63	24.0	6.4936	< .001
		-	CL	Right	15.7936	3.39	24.0	4.6580	0.009
		-	CR	Both	23.6729	3.86	24.0	6.1392	< .001
		-	CR	Left	18.8339	3.77	24.0	4.9942	0.004
	Right	-	CL	Both	21.9904	3.65	24.0	6.0297	< .001
		-	CL	Right	14.1896	2.93	24.0	4.8446	0.006
		-	CR	Both	22.0689	4.21	24.0	5.2439	0.002
		-	CR	Left	17.2300	4.20	24.0	4.1036	0.031
		-	PR	Right	-21.1079	4.86	24.0	-4.3419	0.018
CL	Both	-	CL	Left	-17.3104	3.73	24.0	-4.6396	0.009
		-	CR	Right	-20.7982	3.67	24.0	-5.6685	< .001
		-	PL	Both	-18.0371	3.80	24.0	-4.7488	0.007
		-	PL	Left	-31.5104	4.47	24.0	-7.0567	< .001
		-	PL	Right	-26.3582	3.39	24.0	-7.7780	< .001
		-	PR	Both	-24.4846	4.51	24.0	-5.4280	0.001
		-	PR	Left	-34.6936	5.32	24.0	-6.5158	< .001
		-	PR	Right	-43.0982	5.83	24.0	-7.3926	< .001
	Left	-	CR	Both	17.3889	3.74	24.0	4.6510	0.009
		-	PR	Right	-25.7879	6.14	24.0	-4.1973	0.025
	Right	-	CR	Right	-12.9975	3.11	24.0	-4.1805	0.026
		-	PL	Left	-23.7096	4.58	24.0	-5.1774	0.003
		-	PL	Right	-18.5575	3.40	24.0	-5.4516	0.001
		-	PR	Left	-26.8929	5.39	24.0	-4.9922	0.004
		-	PR	Right	-35.2975	5.49	24.0	-6.4322	< .001
CR	Both	-	CR	Right	-20.8768	4.09	24.0	-5.1024	0.003
		-	PL	Both	-18.1157	4.28	24.0	-4.2360	0.023
		-	PL	Left	-31.5889	4.52	24.0	-6.9950	< .001
		-	PL	Right	-26.4368	3.80	24.0	-6.9497	< .001
		-	PR	Both	-24.5632	4.65	24.0	-5.2841	0.002
		-	PR	Left	-34.7721	5.47	24.0	-6.3550	< .001
		-	PR	Right	-43.1768	5.96	24.0	-7.2477	< .001
	Left	-	PL	Left	-26.7500	4.94	24.0	-5.4122	0.001
		-	PL	Right	-21.5979	4.53	24.0	-4.7647	0.007
		-	PR	Left	-29.9332	5.41	24.0	-5.5289	0.001
		-	PR	Right	-38.3379	6.29	24.0	-6.0921	< .001
PL	Both	-	PR	Right	-25.0611	5.35	24.0	-4.6883	0.008
	Right	-	PR	Right	-16.7400	4.24	24.0	-3.9457	0.044
PR	Both	-	PR	Right	-18.6136	4.26	24.0	-4.3704	0.017

Table S11. RMANOVA for Motor Imagery Alpha band - Post Hoc Comparisons - ROI ∗ Condition. Only statistically significant comparisons are shown.

Within Subjects Effects						
	**Sphericity Correction**	**df**	**Mean Square**	**F**	**p**	
ROI	None	5	1499.7	13.083	< .001	**
	Greenhouse-Geisser	3.01	2495.2	13.083	< .001	**
ROI ∗ Sex	None	5	119.5	1.043	0.396	
	Greenhouse-Geisser	3.01	198.9	1.043	0.379	
ROI ∗ Handedness	None	5	531.3	4.635	< .001	**
	Greenhouse-Geisser	3.01	883.9	4.635	0.005	*
ROI ∗ Sex ∗ Handedness	None	5	34.8	0.303	0.910	
	Greenhouse-Geisser	3.01	57.9	0.303	0.823	
Residual	None	120	114.6			
	Greenhouse-Geisser	72.12	190.7			
Condition	None	2	789.0	6.374	0.004	*
	Greenhouse-Geisser	1.90	829.6	6.374	0.004	*
Condition ∗ Sex	None	2	164.6	1.330	0.274	
	Greenhouse-Geisser	1.90	173.1	1.330	0.274	
Condition ∗ Handedness	None	2	86.9	0.702	0.500	
	Greenhouse-Geisser	1.90	91.4	0.702	0.494	
Condition ∗ Sex ∗ Handedness	None	2	61.2	0.495	0.613	
	Greenhouse-Geisser	1.90	64.4	0.495	0.604	
Residual	None	48	123.8			
	Greenhouse-Geisser	45.65	130.1			
ROI ∗ Condition	None	10	139.8	4.274	< .001	**
	Greenhouse-Geisser	4.43	315.8	4.274	0.002	*
ROI ∗ Condition ∗ Sex	None	10	13.2	0.404	0.944	
	Greenhouse-Geisser	4.43	29.8	0.404	0.824	
ROI ∗ Condition ∗ Handedness	None	10	34.7	1.060	0.394	
	Greenhouse-Geisser	4.43	78.4	1.060	0.383	
ROI ∗ Condition ∗ Sex ∗ Handedness	None	10	24.1	0.738	0.689	
	Greenhouse-Geisser	4.43	54.5	0.738	0.581	
Residual	None	240	32.7			
	Greenhouse-Geisser	106.26	73.9			

Table S12. RMANOVA for Motor Imagery Beta band - Within subjects effects.

Between Subjects Effects					
	**df**	**Mean Square**	**F**	**p**	
Sex	1	159.8	0.1182	0.734	
Handedness	1	5545.9	4.1035	0.054	(*)
Sex ∗ Handedness	1	54.4	0.0403	0.843	
Residual	24	1351.5			

Table S13. RMANOVA for Motor Imagery Beta band - Between subjects effects. The symbol (*) indicates almost significance.

Tests of Sphericity					
	**Mauchly's W**	**p**	**Greenhouse-Geisser ϵ**	**Huynh-Feldt ϵ**	
ROI	0.11648	< .001	0.601	0.697	**
Condition	0.94855	0.545	0.951	1.000	
ROI ∗ Condition	0.00447	< .001	0.443	0.555	**

Table S14. RMANOVA for Motor Imagery Beta band - Tests of sphericity.

Post Hoc Comparisons - ROI $*$ Condition									
**Comparison**									
**ROI**	**Condition**		**ROI**	**Condition**	**Mean Difference**	**SE**	**df**	**t**	$\mathbf{p}_{tukey}$
FL	Both	-	CL	Both	10.9111	1.64	24.0	6.6500	$<.001$
		-	CR	Both	9.5171	1.95	24.0	4.8854	0.005
	Left	-	CL	Both	12.2125	1.99	24.0	6.1364	$<.001$
		-	CL	Left	6.8364	1.69	24.0	4.0400	0.036
		-	CR	Both	10.8186	2.13	24.0	5.0739	0.003
		-	CR	Left	8.7739	2.17	24.0	4.0362	0.036
	Right	-	CL	Both	12.0014	1.90	24.0	6.3308	$<.001$
		-	CL	Left	6.6254	1.57	24.0	4.2320	0.023
		-	CR	Both	10.6075	2.22	24.0	4.7703	0.007
FR	Both	-	CL	Both	11.5646	1.68	24.0	6.9042	$<.001$
		-	CR	Both	10.1707	1.83	24.0	5.5717	0.001
	Left	-	CL	Both	11.6018	1.92	24.0	6.0340	$<.001$
		-	CL	Left	6.2257	1.43	24.0	4.3565	0.018
		-	CR	Both	10.2079	2.00	24.0	5.1043	0.003
		-	CR	Left	8.1632	1.97	24.0	4.1412	0.029
	Right	-	CL	Both	11.9729	2.09	24.0	5.7224	$<.001$
		-	CR	Both	10.5789	2.45	24.0	4.3095	0.020
CL	Both	-	CR	Right	-10.5836	2.28	24.0	-4.6321	0.009
		-	PL	Left	-11.8282	2.36	24.0	-5.0105	0.004
		-	PL	Right	-10.3907	2.30	24.0	-4.5178	0.012
		-	PR	Both	-9.5507	2.16	24.0	-4.4308	0.015
		-	PR	Left	-14.0404	2.30	24.0	-6.1061	$<.001$
		-	PR	Right	-17.7929	2.72	24.0	-6.5526	$<.001$
	Left	-	PR	Right	-12.4168	2.55	24.0	-4.8673	0.005
	Right	-	PR	Right	-14.1943	2.95	24.0	-4.8195	0.006
CR	Both	-	CR	Right	-9.1896	2.18	24.0	-4.2092	0.025
		-	PL	Left	-10.4343	2.37	24.0	-4.3951	0.016
		-	PL	Right	-8.9968	2.31	24.0	-3.8954	0.049
		-	PR	Both	-8.1568	1.71	24.0	-4.7793	0.007
		-	PR	Left	-12.6464	1.79	24.0	-7.0486	$<.001$
		-	PR	Right	-16.3989	2.60	24.0	-6.3185	$<.001$
	Left	-	PR	Left	-10.6018	1.96	24.0	-5.4058	0.002
		-	PR	Right	-14.3543	2.71	24.0	-5.2900	0.002

Table S15. RMANOVA for Motor Imagery Beta band - Post Hoc Comparisons - ROI $*$ Condition. Only statistically significant comparisons are shown.

Post Hoc Comparisons - ROI ∗ Handedness

	Comparison									
**ROI**	**Handedness**		**ROI**	**Handedness**	**Mean Difference**	**SE**	**df**	**t**	$\mathbf{p}_{tukey}$	
FL	left	-	FL	right	10.898	3.029	24.0	3.598	0.051	(*)
		-	FR	left	1.129	0.975	24.0	1.158	0.988	
		-	FR	right	9.760	2.876	24.0	3.393	0.078	
		-	CL	left	9.080	2.113	24.0	4.298	0.010	*
		-	CL	right	19.252	3.781	24.0	5.091	0.002	*
		-	CR	left	7.233	2.256	24.0	3.205	0.114	
		-	CR	right	16.805	3.546	24.0	4.739	0.004	*
		-	PL	left	6.595	2.458	24.0	2.683	0.292	
		-	PL	right	7.890	3.900	24.0	2.023	0.675	
		-	PR	left	3.743	2.380	24.0	1.572	0.903	
		-	PR	right	2.983	3.717	24.0	0.803	0.999	
	right	-	FR	left	-9.769	2.876	24.0	-3.397	0.078	
		-	FR	right	-1.139	0.975	24.0	-1.168	0.987	
		-	CL	left	-1.818	3.781	24.0	-0.481	1.000	
		-	CL	right	8.353	2.113	24.0	3.954	0.023	*
		-	CR	left	-3.665	3.546	24.0	-1.034	0.995	
		-	CR	right	5.906	2.256	24.0	2.618	0.323	
		-	PL	left	-4.303	3.900	24.0	-1.103	0.992	
		-	PL	right	-3.008	2.458	24.0	-1.224	0.981	
		-	PR	left	-7.155	3.717	24.0	-1.925	0.734	
		-	PR	right	-7.915	2.380	24.0	-3.325	0.090	
FR	left	-	FR	right	8.630	2.715	24.0	3.179	0.120	
		-	CL	left	7.951	2.236	24.0	3.556	0.056	
		-	CL	right	18.122	3.660	24.0	4.951	0.002	*
		-	CR	left	6.104	2.289	24.0	2.667	0.299	
		-	CR	right	15.675	3.417	24.0	4.588	0.005	*
		-	PL	left	5.466	2.330	24.0	2.346	0.474	
		-	PL	right	6.761	3.783	24.0	1.787	0.810	
		-	PR	left	2.614	2.395	24.0	1.091	0.992	
		-	PR	right	1.854	3.594	24.0	0.516	1.000	
	right	-	CL	left	-0.679	3.660	24.0	-0.186	1.000	
		-	CL	right	9.492	2.236	24.0	4.246	0.012	*
		-	CR	left	-2.527	3.417	24.0	-0.740	1.000	
		-	CR	right	7.045	2.289	24.0	3.079	0.146	
		-	PL	left	-3.164	3.783	24.0	-0.836	0.999	
		-	PL	right	-1.870	2.330	24.0	-0.802	0.999	
		-	PR	left	-6.017	3.594	24.0	-1.674	0.863	
		-	PR	right	-6.777	2.395	24.0	-2.829	0.229	
CL	left	-	CL	right	10.171	4.407	24.0	2.308	0.497	
		-	CR	left	-1.847	1.649	24.0	-1.120	0.990	
		-	CR	right	7.725	4.207	24.0	1.836	0.784	
		-	PL	left	-2.485	3.042	24.0	-0.817	0.999	
		-	PL	right	-1.190	4.510	24.0	-0.264	1.000	
		-	PR	left	-5.337	2.765	24.0	-1.930	0.731	
		-	PR	right	-6.097	4.352	24.0	-1.401	0.952	
	right	-	CR	left	-12.019	4.207	24.0	-2.857	0.218	
		-	CR	right	-2.447	1.649	24.0	-1.484	0.931	
		-	PL	left	-12.656	4.510	24.0	-2.807	0.238	
		-	PL	right	-11.362	3.042	24.0	-3.736	0.038	
		-	PR	left	-15.509	4.352	24.0	-3.564	0.055	
		-	PR	right	-16.269	2.765	24.0	-5.883	< 001	**
CR	left	-	CR	right	9.572	3.997	24.0	2.395	0.445	
		-	PL	left	-0.637	3.070	24.0	-0.208	1.000	
		-	PL	right	0.657	4.314	24.0	0.152	1.000	
		-	PR	left	-3.490	2.264	24.0	-1.542	0.913	
		-	PR	right	-4.250	4.149	24.0	-1.024	0.995	
	right	-	PL	left	-10.209	4.314	24.0	-2.366	0.462	
		-	PL	right	-8.915	3.070	24.0	-2.904	0.201	
		-	PR	left	-13.062	4.149	24.0	-3.148	0.128	
		-	PR	right	-13.822	2.264	24.0	-6.105	< .001	**
PL	left	-	PL	right	1.295	4.610	24.0	0.281	1.000	
		-	PR	left	-2.853	2.023	24.0	-1.410	0.950	
		-	PR	right	-3.613	4.456	24.0	-0.811	0.999	
	right	-	PR	left	-4.147	4.456	24.0	-0.931	0.998	
		-	PR	right	-4.907	2.023	24.0	-2.426	0.427	
PR	left	-	PR	right	-0.760	4.296	24.0	-0.177	1.000	

Table S16. RMANOVA for Motor Imagery Beta band - Post Hoc Comparisons - ROI ∗ Handedness. The symbol (*) indicates almost significance.
